# A novel jamming phase diagram links tumor invasion to non-equilibrium phase separation

**DOI:** 10.1016/j.isci.2021.103252

**Published:** 2021-10-12

**Authors:** Wenying Kang, Jacopo Ferruzzi, Catalina-Paula Spatarelu, Yu Long Han, Yasha Sharma, Stephan A. Koehler, Jennifer A. Mitchel, Adil Khan, James P. Butler, Darren Roblyer, Muhammad H. Zaman, Jin-Ah Park, Ming Guo, Zi Chen, Adrian F. Pegoraro, Jeffrey J. Fredberg

**Affiliations:** 1 Department of Environmental Science, Harvard T.H. Chan School of Public Health, Boston, MA 02115, USA; 2 Department of Biomedical Engineering, Boston University, Boston, MA 02215, USA; 3 Department of Bioengineering, University of Texas at Dallas, Richardson, TX 75080, USA; 4 Thayer School of Engineering, Dartmouth College, Hanover, NH 03755, USA; 5 Department of Mechanical Engineering, Massachusetts Institute of Technology, Cambridge, MA 02139, USA; 6 Department of Medicine, Brigham and Women’s Hospital and Harvard Medical School, Boston, MA 02115, USA; 7 Howard Hughes Medical Institute, Boston University, Boston, MA 02115, USA; 8 Department of Physics, University of Ottawa, Ottawa, ON K1N 6N5, Canada

**Keywords:** Biophysics, Cancer, Mechanobiology

## Abstract

It is well established that the early malignant tumor invades surrounding extracellular matrix (ECM) in a manner that depends upon material properties of constituent cells, surrounding ECM, and their interactions. Recent studies have established the capacity of the invading tumor spheroids to evolve into coexistent solid-like, fluid-like, and gas-like phases. Using breast cancer cell lines invading into engineered ECM, here we show that the spheroid interior develops spatial and temporal heterogeneities in material phase which, depending upon cell type and matrix density, ultimately result in a variety of phase separation patterns at the invasive front. Using a computational approach, we further show that these patterns are captured by a novel jamming phase diagram. We suggest that non-equilibrium phase separation based upon jamming and unjamming transitions may provide a unifying physical picture to describe cellular migratory dynamics within, and invasion from, a tumor.

## Highlights

• Tumor spheroids invading into a 3D matrix exhibit coexistent material phases
• Invasion leads to spatiotemporal heterogeneities consistent with jamming dynamics
• Phase separation patterns are captured by a model-based jamming phase diagram
• Effective thermodynamic variables *T*_*eff*_ and *P*_*conf*_ capture cell and matrix properties

## Graphical abstract

**Figure:**
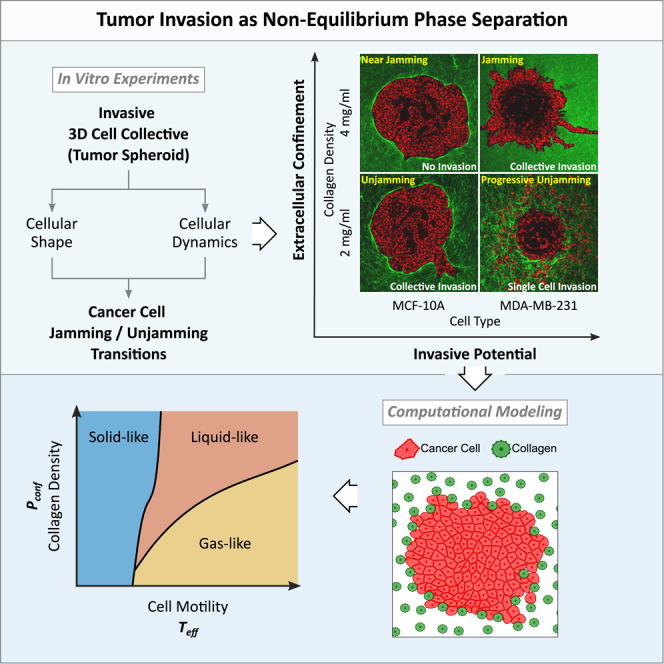

## Introduction

Invasion of cancer cells from a primary tumor into surrounding tissue is a process wherein migration phenotypes can differ dramatically depending on the properties of the cells and those of the surrounding ECM (Lautscham et al., 2015). It is well established that changes from one migratory phenotype to another depend upon a variety of factors that are cell specific, matrix specific, and interactive (Friedl et al., 2012; Lautscham et al., 2015). Current understanding of tumor dynamics begins with a core set of genetic alterations followed by driver mutations, evolution, competition, and resulting sub-clonal heterogeneity within the tumor mass (Ghajar and Bissell, 2016; Waclaw et al., 2015). Cell invasion and escape from the primary tumor are usually thought to require the epithelial-to-mesenchymal transition (EMT) and associated degradation of cell-cell adhesion through loss of E-cadherin. EMT transforms nominally non-migratory epithelial cells into highly migratory mesenchymal cells that can invade individually into surrounding ECM (Zhang and Weinberg, 2018). In multiple models of breast cancer, paradoxically, metastasis requires E-cadherin nevertheless (Padmanaban et al., 2019). A unifying physical picture that describes invasion of cancer cells, either as single cells, multicellular collectives, or the transition between them is currently lacking.

From tumors of epithelial origin, cells typically invade collectively as multicellular strands, sheets, or clusters (Clark and Vignjevic, 2015; Friedl and Gilmour, 2009; Friedl et al., 2012; Khalil et al., 2017). Mesenchymal clusters under confinement can also display collective migration and invasion despite their lack of cell-cell adhesions (Haeger et al., 2014). As regards the physics of collective cellular migration in wound healing, development, and cancer invasion, recent evidence implicates the transition from a solid-like jammed phase to a fluid-like unjammed phase by means of the unjamming transition, or UJT (Angelini et al., 2011; Park et al., 2015; Valencia et al., 2015; Oswald et al., 2017; Palamidessi et al., 2019; Ilina et al., 2020). Experimental work using tumor spheroids further suggests that the spheroid core approximates a jammed, solid-like phase in which cellular shapes tend to be rounded and cellular motions tend to be limited, intermittent, and caged by surrounding cells (Valencia et al., 2015; Palamidessi et al., 2019). The spheroid periphery, by contrast, approaches an unjammed, fluid-like phase in which cell shapes tend to become elongated and cellular motions tend to become larger, more cooperative, more persistent, and sometimes rotational (Han et al., 2020). Moreover, compared with cells in the spheroid core, cells at the spheroid periphery and invasive branches tend to become systematically softer, larger, longer, and more dynamic; these mechanical changes arise in part from supracellular fluid flow through gap junctions, suppression of which delays transition to an invasive phenotype (Han et al., 2020). At the molecular level, increased levels of the small GTPase RAB5A and associated hyper-activation of the kinase ERK1/2 and phosphorylation of the actin nucleator WAVE2 have been implicated in cell unjamming (Palamidessi et al., 2019). Furthermore, the first genome-wide transcriptomic analysis of biological processes that underlie UJT were carried out for the case of human primary bronchial epithelial cells (HBECs) (De Marzio et al., 2021). Among many other factors, that analysis supports the involvement of cell-ECM adhesions, actomyosin reorganization, activation of ERK and JNK pathways, and downregulation of morphogenetic and developmental pathways (De Marzio et al., 2021). While that analysis is particular to HBECs, it highlights the fact that the UJT program comprises a coordinated time-dependent interplay of many signaling processes. In HBECs, moreover, UJT is distinct from—and can occur independently of—EMT (Mitchel et al., 2020). This complex network of interconnected molecular events combines to yield the UJT, which is itself a complex biophysical process resulting from changes to the cell and altered interactions with the surrounding microenvironment.

Using a variety of breast cancer models, Ilina et al. (2020) recently demonstrated that cell-cell adhesions and mechanical confinement by the surrounding ECM contribute to collective invasion through cell density regulation. They sketched a hypothetical jamming phase diagram in which the interplay between these two factors progressively unjams the tumor from a solid-like non-invasive phase to a fluid-like invading collective, and, finally, to individualized cells that scatter like a gas. Hence, progressive unjamming of a cellular collective by means of the UJT seems to be a major physical route to invasion (Oswald et al., 2017; Palamidessi et al., 2019; Ilina et al., 2020). Using multicellular breast cancer spheroids invading into a three-dimensional (3D) ECM, here we investigate the evolution of distinct material phases within the invasive cellular collective. By quantifying the distribution of cell shapes, packing, and migratory dynamics in both space and time during 3D invasion, we find strong evidence for coexistence of—and transition between—solid-like, fluid-like, and gas-like collective cellular phases, thus confirming earlier findings (Ilina et al., 2020). Unlike earlier studies, however, we show further that depending on cell type and ECM density, routes toward collective invasion can involve not only the unjamming transition, but also the jamming transition. Moreover, a hybrid computational model recapitulates these collective behaviors and results in a novel jamming phase diagram. We further propose that this jamming phase diagram may be governed by a small set of ‘effective’ thermodynamic variables, which provide a unifying framework to study the biophysical mechanisms of 3D tumor invasion.

## Results

### In the MCF-10A micro-spheroid, the core approaches a jammed, solid-like phase

#### Structural signatures of unjamming

**Figure 1:**
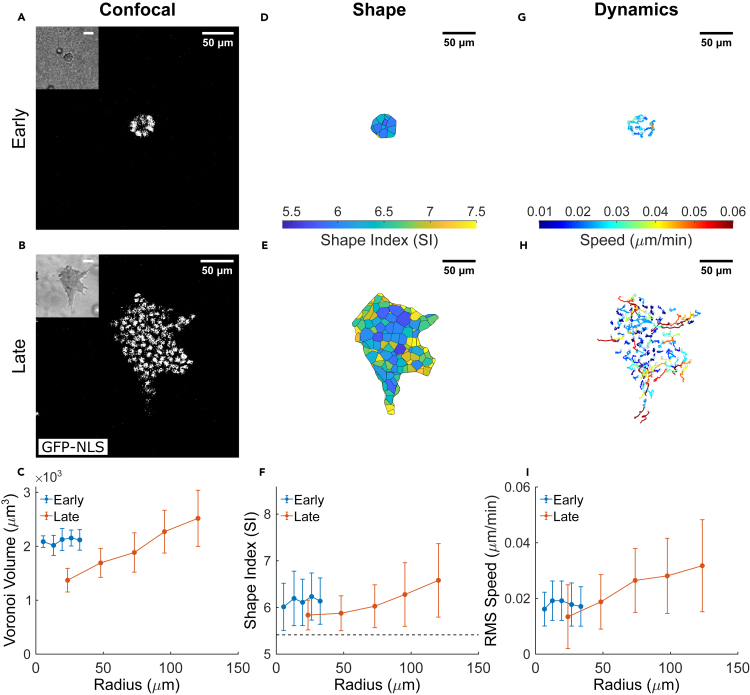
The MCF-10A micro-spheroid exhibits a jammed solid-like core and an unjammed fluid-like periphery (A and B) Equatorial cross-sections of confocal microscopy images show cell nuclei distribution within micro-spheroids grown from GFP-NLS labeled MCF-10A cells at distinct stages of spheroid evolution: early stage (days 3–5; A) and late stage (days 7–10; B). Micro-spheroids at late stage are much larger compared to the early stage, and show clear invasive protrusions that extend into the ECM. Corresponding cross-sections of bright-field microscopy images outline the micro-spheroid boundary (inset), and are used to generate bounded Voronoi tessellation to estimate cell shape. (C) In late-stage spheroids, but not early-stage spheroids, cell volumes obtained by tessellation of nuclear centroids increase with increasing radial position. This result is consistent with previous observations in this model system and attributed to an increase in intra-tumor compressive stress (Han et al., 2020) (D and E) The corresponding cell shapes are shown as 2D cross-sections, color-coded according to their respective 3D Shape Index (SI). Cell SIs exhibit more variability in the late-, than early-stage spheroid. (F) Compared to early stage, cells in the late-stage spheroid core have smaller average SI. In the late-stage spheroid, but not early-stage, SI increased with increasing radial position within the spheroid. This is suggestive of the development of a jammed solid-like core and an unjammed fluid-like periphery. The horizontal dashed line indicates the SI threshold for solid to fluid transition, where proximity away from the threshold (SI > 5.4) suggests transition toward a more fluid-like phase (Merkel and Manning, 2018). (G and H) 2D projections of 3D nuclear trajectories tracked over 8 h reveal that within the early-stage spheroid cell migration is fairly homogeneous, whereas in the late-stage spheroid, migratory patterns become highly dynamic. Nuclear trajectories are color-coded according to average migratory speed of the cell over the observation window. (I) Compared to the homogeneous cell dynamics in the early-stage spheroid, cells in the late-stage spheroid develop a positive radial gradient in migratory dynamics. Consequently, less motile cells are located in the jammed core while more motile cells are located in the unjammed periphery. Data for radial distributions are presented as mean ± STD (n = 5 for both early and late stage spheroids).

As a simplified model of an invasive carcinoma, we used MCF-10A epithelial cells (Figure S1) embedded within an engineered interpenetrating network of Matrigel and alginate (Chaudhuri et al., 2014; Han et al., 2020) (STAR Methods). In these non-malignant cells, ECM stiffness by itself is sufficient to induce a malignant phenotype (Chaudhuri et al., 2014). Here, we varied the concentration of alginate without changing the concentration of Matrigel, thus tuning the extracellular environment to a stiffness comparable to malignant breast cancer tissue. Within such matrices, MCF-10A cells spontaneously proliferate, form a micro-spheroid and, over time, invade the surrounding ECM (Figures 1A and 1B). Previous studies have found that this spontaneous invasive behavior cannot be explained by differences in spheroid size or cell differentiation over time, but rather is attributed to physical changes in cell properties (Han et al., 2020). We hypothesized, therefore, that the observed collective invasion may be an emergent property associated with cell unjamming. For this reason, we examined evolution of the micro-spheroid at an early stage (days 3–5), when the spheroid is typically 30μm in radius and contains 46 ± 21 cells, and at a later stage (days 7–10), when asymmetric invasive protrusions extend up to 120 μm from the spheroid center and the spheroid contains 169 ± 47 cells.

To quantify structural characteristics and packing of constituent cells, we identified nuclear locations from 3D confocal microscopy of cells transfected with green fluorescent protein tagged with nuclear localization signal (GFP-NLS). The spheroid boundary was identified from corresponding bright-field images (Figures 1A and 1B, insets). Using a bounded Voronoi approach based upon nuclear centroids, we then tessellated the space at each stage of spheroid growth and calculated for each cell the surface area, S, and the volume, V (STAR Methods, Figure S2). Consistent with previous reports, cell volumes and nuclear volumes varied systematically throughout the spheroid and co-varied linearly (Figure S3) (Jovtchev et al., 2006; Guo et al., 2017; Han et al., 2020). In the late stage micro-spheroid, in particular, we found that cell volumes increased with radial position (Figure 1C) and cell number densities correspondingly decreased. These gradients have been observed previously and linked to supracellular fluid flow driven by frequently reported gradients of intra-tumor compressive stress (Han et al., 2020). Compressive stress within tumors is known to become large enough to collapse intra-tumor blood vessels and to increase the metastatic potential of cancer cells (Tse et al., 2012; Stylianopoulos et al., 2013).

We calculated for each cell a non-dimensional shape index (SI), given as the normalized surface area, SI = S/V^2/3^, which can then be used as a structural signature of the degree of local unjamming and fluidization of the collective (Park et al., 2015; Bi et al., 2016; Merkel and Manning, 2018) (STAR Methods; Figure S2). In two-dimensions (2D), cell shapes become progressively more elongated and more variable as the cell layer becomes progressively more unjammed (Park et al., 2015; Atia et al., 2018; Merkel and Manning, 2018). This change is seen to occur in a continuous fashion, akin to jamming or glass transitions, as discussed below, with no discrete, structurally-distinct phase boundary being evident. In the early-stage micro-spheroid, SIs were distributed homogenously with a mean of 6.01 ± 0.51 (Figure 1D). In the late-stage micro-spheroid, however, SIs exhibited a clear positive radial gradient (Figure 1E). Near the spheroid periphery SI was 6.6 ± 0.79, which is greater than at the center where SI was 5.84 ± 0.32 (p<0.01). Values of the SI at the center of late-stage spheroids approached, though never fully reached, the critical value of 5.4 predicted by a recent 3D Voronoi model for cellular jamming (Merkel and Manning, 2018). Accordingly, MCF-10A micro-spheroids seem to exist in a liquid phase which is homogeneous at the non-invasive early stage, while it develops radial heterogeneities consistent with a glassy transition at the invasive late stage. These regional differences in SI are indicative of the spheroid center being in a material phase in closer proximity to a jamming transition, while the spheroid periphery is a material phase that is further removed from a jamming transition (Figure 1F). At a late stage, therefore, the spheroid center tends to become more solid-like while the periphery tends to become more fluid-like. We also determined for each cell the 3D principal aspect ratios (AR_1_, AR_2_ and AR_3_), which confirmed the radial trend toward unjamming suggested by the SI (Figure S4). The distributions of SI (***x***) were described by a k-gamma probability density function, $\text{PDF}\left(x;k\right)=k^{k}x^{k-1}e^{-kx}/\text{Γ}\left(k\right)$, where one interpretation for the parameter k is the interaction range between neighbors in influencing particle packing (Figure S4). In all cases the distributions of SI variation from cell-to-cell conformed to a *k-gamma* distribution (Figure S4). In living systems, inert systems, and computational models, the *k-gamma* distribution has been thought to be yet another structural signature of granular systems approaching a jammed packing (Aste and Di Matteo, 2008; Park et al., 2015; Atia et al., 2018). Using maximum likelihood estimation across all spheroid preparations, the average value of *k* was 10.2 ± 0.1, within the range of values previously reported in inert jammed 3D systems (Aste and Di Matteo, 2008). We found little variation in *k* between stages of spheroid evolution (Figure S4). This finding is reminiscent of those reported in quasi 2D cell layers (Atia et al., 2018), where over a wide range of cell types, *in vivo* and *in vitro*, different underlying pathological conditions, and even different species, *k* was found to fall into a narrow range between 1.9 and 2.5. It remains an open question as to why *k* seems to depend so strongly on dimensionality of the system (i.e., 2D versus 3D) but relatively little on the nature of constituent particles.

#### Migratory signatures of unjamming

From image stacks acquired over 8 h of micro-spheroid growth and cellular migration, we tracked for each cell the nuclear trajectory (STAR Methods). In the early-stage micro-spheroid there was no spatial gradient in cellular migratory speed, whereas in the late-stage micro-spheroid the migratory speed increased systematically from the core to the periphery (Figure 1I). Cellular motions in the late-stage micro-spheroid core were small, sub-diffusive and thus showed evidence of caging (Figure S5). In contrast, cellular motions in the late-stage micro-spheroid periphery were larger, super-diffusive, and thus showed no evidence of caging, consistent with reports from Valencia et al. (2015). As a measure of tissue fluidity we calculated the relaxation rate of a self-overlap parameter (STAR Methods) which quantifies the local cellular rearrangement rate after accounting for global spheroid motion. Such relaxation rate shows a positive radial gradient (Figure S5C) similar to that reported by Palamidessi et al. (2019), and suggests that the increased motility at the late-stage micro-spheroid periphery is accompanied by frequent local cell rearrangements, thus indicating an increased tissue fluidity. Together, these dynamic features suggest that the increase in cell SIs at the invasive periphery reflects an increase in tissue fluidity in a manner that is consistent with predictions from the static theory of 3D jamming (Merkel and Manning, 2018). These structural and migratory behaviors further support the interpretation that the center of the more mature spheroid tends to become more jammed and solid-like, whereas the periphery tends to become more unjammed and fluid-like (Valencia et al., 2015; Palamidessi et al., 2019; Han et al., 2020).

### In the MCF-10A macro-spheroid, the periphery invades as a locally unjammed fluid-like phase

**Figure 2:**
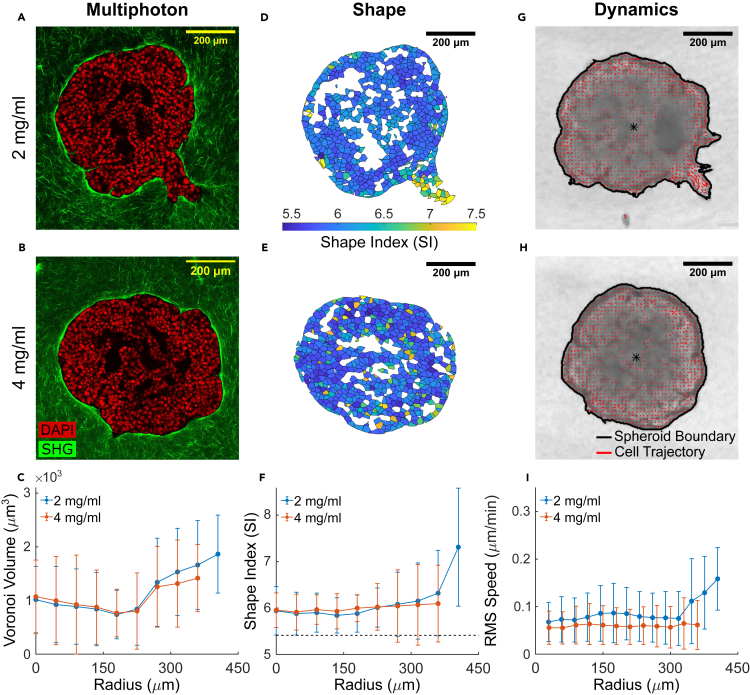
In a manner dependent on collagen concentration, the MCF-10A macro-spheroid locally unjams and fluidizes at the periphery during collective invasion (A and B) Representative equatorial cross-sections of multiphoton images show MCF-10A macro-spheroid behavior when embedded in either 2 (low density) or 4 mg/mL (high density) collagen for 48 h, with DAPI-stained cell nuclei shown in red and collagen fibers from SHG shown in green. In low density collagen (A), the spheroid develops collective invasive protrusions, while in high density collagen (B), no invasion is observed. Cell-free voids (black) are due to matrigel used to promote spheroid formation (STAR Methods, Figure S1); cells immediately neighboring this cell-free region are excluded from subsequent structural analysis (STAR Methods, Figure S2). (C) Similar to observations from the MCF-10A micro-spheroids, Voronoi cell volumes increased from the macro-spheroid core to the periphery. In contrast to the micro-spheroids, average cell volumes from macro-spheroids cultured at both collagen densities are smaller, and suggest that cells in the macro-spheroid experience greater compressive stress (cf. Figure 1C). (D and E) The corresponding cell shapes are shown as 2D cross-sections, color-coded according to their respective 3D Shape Index (SI). Increased and more variable SIs are localized in the region of the spheroid periphery that undergoes collective invasion (D). On the other hand, SIs remain narrowly distributed, in the rest of the spheroid periphery and in the core regardless of collagen density. (F) In fact, SIs are homogeneously distributed near the threshold for solid-fluid transition (horizontal dashed line indicates solid-fluid transition point at SI = 5.4 (Merkel and Manning, 2018)). SIs increased only at the invasive protrusions suggest localized unjamming and fluidization is associated with invasion. (G and H) Representative DIC images are shown for MCF-10A macro-spheroids cultured in 2 and 4 mg/mL collagen, with cell migratory trajectories (from optical flow, STAR Methods) superimposed in red. Longer trajectories are observed at the collectively invading regions (G). The spheroid boundaries are outlined in black. The entire DIC time-lapse video capturing the dynamics of invasion over 48 h is shown in Video S1. (I) Radial distributions of average cell migratory speed quantified for the final 8-h observation window (40–48 h) conform to expectations from cell shapes. In both collagen densities, migratory speed is homogenously low in the spheroid core, and increased only at sites of localized invasive protrusions. Data for radial distributions are presented as mean ± STD (n = 3 for both 2 and 4 mg/mL spheroids).

To assess the generality of these results, we examined invasion patterns and cell unjamming signatures in macro-spheroids embedded in matrices spanning a range of collagen densities. Compared to the micro-spheroids described above, these macro-spheroids were larger (extending to a radius of approximately 450μm from the spheroid center) and contained roughly 10-fold to 100-fold as many cells. To form a macro-spheroid, we cultured MCF-10A cells on a low attachment substrate in the presence of a small volume fraction of Matrigel and allowed the cells to coalesce into a cluster over a period of 48 h (STAR Methods, Figure S6). This cluster was then embedded into a self-assembling network of rat-tail collagen I fibrils at either low (2 mg/mL) or high (4 mg/mL) concentration. Using differential interference contrast (DIC) microscopy, these macro-spheroids were imaged continuously as constituent cells proliferated, remodeled the matrix, and initiated invasion. We then used optical clearing (Susaki et al., 2015) and multiphoton microscopy (Karrobi et al., 2019) to obtain stacked images of DAPI-stained nuclei and used second harmonic generation (SHG) signal to obtain images of surrounding collagen (Figures 2A and 2B). Within the macro-spheroid, cell-free voids were frequently observed due to the presence of Matrigel (Figure S6). In these cases, cell shape quantification was restricted to those cells that were either fully surrounded by neighboring cells and/or collagen (STAR Methods, Figure S2). To allow for comparison between radial distributions of cell shape and migratory dynamics, the latter was assessed using optical flow analysis (Vig et al., 2016) of DIC images (STAR Methods, Video S1) for the final 8-h period (40–48 h).

**Figure 3:**
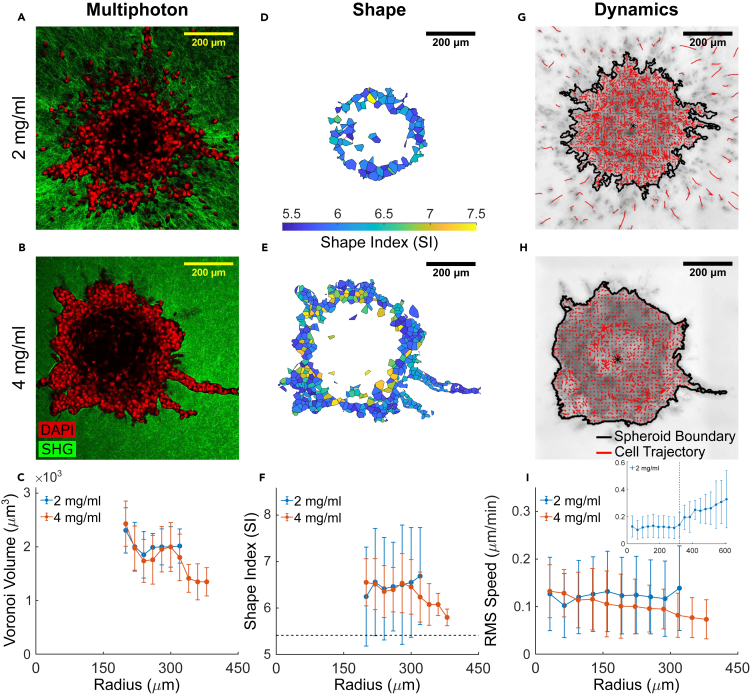
The metastatic MDA-MB-231 spheroid exhibits an unjammed fluid-like phase and undergoes drastically different patterns of invasion depending on collagen concentration (A and B) Representative equatorial cross-sections of multiphoton images show MDA-MB-231 macro-spheroids exhibiting distinct invasion patterns when embedded in low density (2 mg/mL) versus high density (4 mg/mL) collagen for 48 h. DAPI-stained cell nuclei are shown in red and collagen fibers from SHG are shown in green. In low density collagen (A), these metastatic cells scatter from the spheroid core as individual, gas-like particles. Conversely, in high density collagen (B), single-cell dominant scattering is subdued and invasion is in the form of collective, fluid-like protrusions. We note that the center of MDA-MB-231 spheroids is devoid of cells, as confirmed by staining of histological cross-sections (Figure S6), and thus result in a hollow shell of highly motile cells rather than a nearly solid spherical structure. Only cells that remain part of the collective are included in the structural analyses (STAR Methods, Figure S2), hence the absence of data for the first 200 μm of the associated radial distributions. (C) Average Voronoi volumes suggest that MDA-MB-231 cells have larger volumes with respect to their MCF-10A counterparts (cf. Figure 2C). In 2 mg/mL collagen, cell volumes remain roughly independent of radial position. In 4 mg/mL collagen, instead, cell volumes show a decreasing radial gradient. This decrease in cell volume from the spheroid core to the invasive protrusion suggests elevated stress in invading cells from confinement by the collagen matrix. (D and E) The corresponding cell shapes are shown as 2D cross-sections, color-coded according to their respective 3D Shape Index (SI). Regardless of collagen concentration, cells from MDA-MB-231 spheroids display higher SI with respect to MCF-10A spheroids (cf. Figures 2D and 2E). (F) Radial distribution of average SI values is consistent with an unjammed fluid-like phase (horizontal dashed line indicates solid-fluid transition point at SI = 5.4 (Merkel and Manning, 2018)). In high density collagen, a radially decreasing gradient in SI suggests that cells jam while invading collectively under matrix confinement. (G and H) Representative DIC images are shown for MDA-MB-231 macro-spheroids cultured in 2 and 4 mg/mL collagen, with cell migratory trajectories (from optical flow, STAR Methods) superimposed in red. The spheroid boundaries are outlined in black. The entire DIC time-lapse video capturing the dynamics of invasion over 48 h is shown in Video S1. Cell dynamics mirrors structural signatures of cell jamming/unjamming. (I) Radial distributions of RMS speed quantified for the last 8-h observation window (40–48 h) show that cells in MDA-MB-231 macro-spheroids have homogeneously higher speeds with respect to MCF-10A spheroids (cf. Figure 2I) and are thus more fluid-like. In low density collagen, cell speed increases further as soon as cells detach from the spheroid and invade as single, gas-like particles (inset, where the radial position of the spheroid boundary is marked by a dashed vertical line). This observation supports the proposed analogy of fluid-to-gas transition. In high density collagen, RMS speed decrease radially with collective invasion, and is supportive of a fluid-to-solid transition due to confinement-induced jamming (Haeger et al., 2014). Data for radial distributions are presented as mean ± STD (n = 3 for both 2 and 4 mg/mL spheroids).

**Figure 4:**
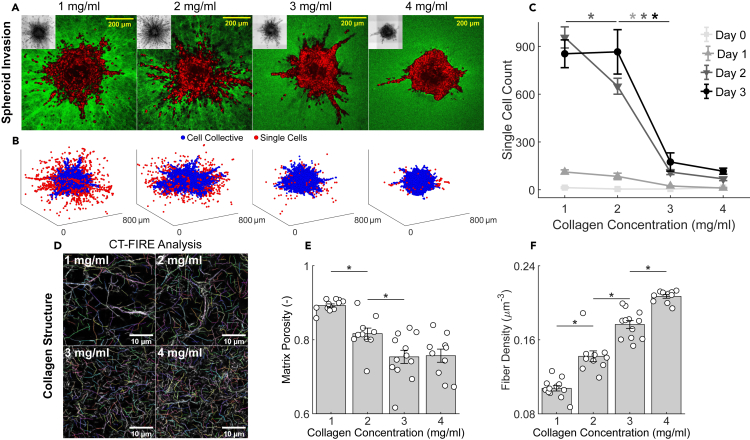
Collagen fiber density is associated with a sudden switch in MDA-MB-231 invasive phenotype (A) Representative equatorial cross-sections of multiphoton images show MDA-MB-231 spheroids after 3 days of invasion in graded collagen concentrations (1–4 mg/mL) along with the associated DIC minimum intensity projections (insets). Single-cell migration is observed primarily in 1 and 2 mg/mL while collective migration is observed primarily in 3 and 4 mg/mL. (B) Corresponding 3D rendering of cell nuclei distributions identified from automated analysis of multiphoton image stacks (STAR Methods). Nuclei are color-coded based on whether they remain within the cell collective (blue) or are detected as single cells (red). (C) Immediately after embedding in collagen (day 0), all cells are part of the multicellular collective with no invasion at any collagen density. As the spheroid evolves over time (days 1, 2 and 3), a striking gas-like phase and corresponding single cell escape progressively emerged at lower collagen concentrations (1 and 2 mg/mL) but not higher collagen concentrations (3 and 4 mg/mL). By day 3, a switch-like biphasic reduction in the number of single invading cells emerged when collagen concentration was increased from 2 to 3 mg/mL. The temporal evolution of single cell invasion as a function of collagen concentration supports the existence of criticality between 2 and 3 mg/mL, at which point the invasive phenotype switches abruptly from single to collective invasion. Single cell counting data are shown from days 0–1–2–3 and collagen concentrations of 1–2–3–4 mg/mL, n = 3 per group, except for day 0–1 mg/mL(n = 2) and day 2–2 mg/mL (n = 9). The significance of differences due to collagen concentration and time were quantified using a one-way ANOVA and post-hoc pairwise comparisons with Bonferroni correction. Statistical significance was achieved between 1 and 2 mg/mL at day 2 (p < 0.05), and between 2 and 3 mg/mL at days 1 (p < 0.05), 2 (p < 0.01), and 3 (p < 0.01), while no significant differences were observed between 3 and 4 mg/mL. We examined whether this transition is due to differences in collagen structure. (D) High-resolution multiphoton images show representative acellular collagen networks at 1 to 4mg/mL, with individually segmented fibers from CT-FIRE analysis (Bredfeldt et al., 2014) as indicated by different colors. (E and F) Matrix porosity shows a gradual decrease with collagen concentration but is undistinguishable between 3 and 4 mg/mL (E), while fiber density displays a consistent increase with collagen concentration (F) which mirrors the increase in bulk shear modulus (Figure S8). Microstructural data are shown from 1 mg/mL (n = 12), 2 mg/mL (n = 10), 3 mg/mL (n = 12), and 4 mg/mL (n = 12) collagen gels. All data are presented as mean ± SEM and ∗ indicates statistical significance at p < 0.05.

Video S1. Representative DIC time-lapse movies capture the migratory dynamics exhibited over the course of 48 h by MCF-10A and MDA-MB-231 spheroids invading in 2 and 4 mg/mL collagen, related to Figures 2–4

At the lower collagen density (2 mg/mL), the MCF-10A macro-spheroids invaded collectively in the form of continuous invasive protrusions and branches (Figure 2A). By contrast, at the higher collagen density (4 mg/mL), no invasion was observed (Figure 2B). Much as in the case of the micro-spheroids, in these macro-spheroids cell volumes were smaller near the core compared to the periphery (Figure 2C). Overall, however, the macro-spheroids exhibited smaller cell volumes than did the micro-spheroids, thereby suggestive of greater compressive stresses in macro-spheroids (Khavari and Ehrlicher, 2019; Han et al., 2020). Regardless of changes in collagen density, the spheroid core displayed SIs that were homogeneous and small, thus indicating proximity to a solid-like jammed phase. By contrast, in low collagen density the collectively invading regions displayed increased and more variable SIs, indicating localized unjamming and progression toward a more fluid-like unjammed phase (Figures 2D–2F). Migratory dynamics conformed to expectations from cell shapes. After correction for spheroid growth (STAR Methods), migratory speed for cells in low density collagen increased mainly in those regions that had larger SIs and were collectively migrating within invasive branches (Figures 2G and 2H). In the epithelial monolayer (Angelini et al., 2011; Czajkowski et al., 2019) and during 3D spheroid growth (Malmi-Kakkada et al., 2018), previous studies have emphasized tissue unjamming and resultant fluidization through the action of cell proliferation. Nevertheless, proliferation seems an unlikely source of the regional unjamming reported here. In our model systems, in fact, changes in collagen density impacted neither spheroid growth nor cell proliferation (Table S1). Instead, we observed larger and more variable cell shapes and faster dynamics restricted to sites where collective invasion occurred.

### In MDA-MB-231 macro-spheroids, the invasive phenotype switches abruptly as a function of ECM density

Localized fluidization of an epithelial cell collective during invasion, such as occurs in the MCF-10A spheroid, involves two main phases: solid-like and fluid-like. To account for invasion via a gas-like phase corresponding to individually migrating cells after EMT, as in Ilina et al. (2020), we performed similar experiments using the post-metastatic cell line, MDA-MB-231. These cells express mesenchymal markers including high vimentin and low E-Cadherin (Liu et al., 2015) (Figure S1), exhibit enriched expression in migration-relevant genes (Lehmann et al., 2011), and form tumors that lead to poor prognosis (Dent et al., 2007). Because spheroid formation in these cells is mediated by integrin β1 adhesion with no cadherin involvement (Ivascu and Kubbies, 2007), these cells form macro-spheroids only in the presence of ECM proteins. Therefore, we formed MDA-MB-231 spheroids by adding 2.5% Matrigel to the cell suspension (Ivascu and Kubbies, 2006) (STAR Methods). For ease of comparison, both MCF-10A and MDA-MB-231 cells were allowed to aggregate using Matrigel, which resulted in macro-spheroids of comparable size (Figure S6). MDA-MB-231 spheroids were composed of approximately 1000–5000 cells and displayed a cell-free core occupied by ECM proteins (Figures 3 and S6). Restricting our structural analysis to the cells remaining within the continuous tumor mass and fully surrounded by neighboring cells and/or collagen, MDA-MB-231 cells had larger volumes with respect to MCF-10A cells after 48 h of culture and invasion (Figures 2C and 3C), larger SIs (Figures 2F and 3F), and higher motility regardless of collagen concentration (Figures 2I and 3I). Therefore, compared to MCF-10A spheroids, cells from post-metastatic MDA-MB-231 spheroids seem to exist in a more motile, fluid-like unjammed phase.

Within the MDA-MB-231 macro-spheroid in lower density collagen (2 mg/mL), cell volumes, SIs, and migratory dynamics were nearly homogeneous (Figures 3C, 3F, and 3I). At the spheroid periphery, MDA-MB-231 cells further unjammed by rapidly detaching from the cell collective, reminiscent of evaporating gas particles, and thereafter invaded as single cells along aligned collagen fibers in a highly dynamic manner (Figure 3I, insert and Video S1). To our surprise, in higher density collagen (4 mg/mL), cell volumes, SIs, and dynamics displayed a radially decreasing trend (Figures 3E and 3F), opposite to that observed in MCF-10A spheroids. Again, migratory dynamics changed in concert with changes in cell shapes (Figure 3I and Video S1). While in lower density collagen migratory speed was homogenously distributed within the tumor mass, in higher density collagen one could observe a consistent slow-down of migratory dynamics within the invasive protrusions, consistent with a jamming (i.e., fluid-to-solid) transition. We confirmed the presence of such jamming transition by monitoring temporal changes in cell shapes and migratory dynamics via time-lapse experiments (Figure S7). Under conditions of greater confinement by the ECM, the number of individualized cells decreased (Figures S7A and S7B) and—as collective branches invaded from the spheroid into the matrix—both SIs and cell speeds decreased in concert (Figure S7C). In addition to the kinetic slow-down, in high density collagen we also found that spatial velocity correlations at the invasive branch were significantly higher with respect to the spheroid core (Figure S7D). Such transition toward slower but more cooperative motions at the invasive front was not detected in spheroids invading in low density collagen where we found higher cell motility but poor alignment between neighboring velocity vectors (Figure S7D), similar to the active fluid phase described previously (Ilina et al., 2020). Moreover, the periphery of spheroids invading into high density collagen displayed primarily radial motions, as opposed to the core where tangential speeds were more prominent (Figure S7E). Overall, the dynamics of collectively invading MDA-MB-231 spheroids reveals that fast but uncorrelated random motions at the spheroid core slow down and the corresponding trajectories become more aligned at the invasive periphery. The higher alignment of velocity vectors at the invasive branches is accompanied by significantly reduced speed and reduction in both cell volumes and cell shape indices, thus supporting the presence of a confinement-induced jamming transition. Therefore, depending on collagen concentration, liquid-like MDA-MB-231 spheroids invade while undergoing liquid-to-gas unjamming (in 2 mg/mL) or progressive re-jamming (in 4 mg/mL), respectively representing single and collective modes of cell migration.

But how does the transition between these different modes of invasion occur as a function of ECM confinement? Using graded concentrations of collagen (1–4 mg/mL), we tracked over time the number of single MDA-MB-231 cells that had detached from the continuous primary spheroid mass, escaped that spheroid, and invaded in a gas-like fashion into the ECM. The number of such single invading cells was found to be not only time-dependent (Figure S7) but, more importantly, dependent on collagen concentration (Figures 4A–4C). On day 0 no cell escape was evident at any collagen concentration; immediately after embedding in collagen, all cells remained within the spheroid. On day 1, a modest level of cell escape became evident at lower collagen concentrations (1 and 2 mg/mL) but not at higher concentrations. On day 2, the number of detached invading cells became much larger and highly sensitive to collagen concentration. By day 3, remarkably, the number of detached invading cells stabilized into a striking biphasic switch-like dependence on collagen concentration. The collagen concentration demarking this step-like transition for MDA-MB-231 spheroids fell between 2 and 3 mg/mL. High-resolution multiphoton microscopy imaging at these graded collagen concentrations (Figure 4D) revealed that as collagen concentration increases matrix porosity decreases and displays a plateau between 3 and 4 mg/mL (Figure 4E), while fiber density increases steadily (Figure 4F). In addition, biomechanical characterization of collagen matrices suggests that such microstructural changes impact mesoscale mechanics, as assessed via unconfined compression at these graded collagen concentrations (Figure S8). We found that the bulk shear modulus increases steadily for increasing collagen concentrations (Figure S8), thus suggesting that higher extracellular stresses develop with increasing collagen fiber density.

Overall, these findings support the possibility of the existence of a critical collagen density at which MDA-MB-231 cells at the spheroid periphery transition in an almost switch-like fashion between distinct modes of invasion. Under lower matrix confinement, unjammed cells tend to invade individually as single cells or discrete cell clusters in a gas-like fashion. Under higher matrix confinement, however, these highly motile cells progressively slow at the spheroid periphery and within invading protrusions they tend to re-jam. For the invading cellular collective, these observations support the interpretation that high density collagen promotes steric hindrance and an associated confinement-induced re-jamming (Wolf et al., 2013; Haeger et al., 2014). These events likely depend upon active remodeling of ECM by metalloproteases (Haeger et al., 2014), cell generated traction forces (Provenzano et al., 2006; Steinwachs et al., 2016; Kim et al., 2020), and the manner in which these tractions act to align collagen fibers (Provenzano et al., 2006). The invasive behaviors and material states observed from micro- and macro-spheroids of MCF-10A and MDA-MB-231 cells are summarized in Table 1.

**Table 1:** Across different spheroid systems, cell types, and experimental conditions, drastic changes in invasive phenotypes are reflected by transitions between material phases

Experimental conditions			Measured trends					Observations			Reference
Spheroid system	Cell type	Condition	Region	Shape Index	Motility	Cell volume	Radial gradient	Invasion	Invasive modality	Material phase	
**Micro** *n*_*cells*_ ∼ O(10^1^-10^2^)	MCF-10A	Early Stage	c	–	–	–	No	No	–	Liquid	Figure 1^a^
			p	–	–	–					
		Late Stage	c	↓	↓	↓	Yes	Yes	Collective	Solid → Liquid	
			p	↑	↑	↑					
**Macro** *n*_*cells*_ ∼ O(10^3^-10^4^)	MCF-10A	Low ECM Density	c	–	–	–	Yes	Yes	Collective	Solid → Liquid	Figure 2^b^
			p	↑	↑	↑					
		High ECM Density	c	–	–	–	No^d^	No	–	Solid	
			p	–	–	↑					
	MDA-MB-231	Low ECM Density	c	↑↑	↑↑	↑	No	Yes	Single Cell	Liquid →Gas	Figures 3 and 4^c^
			P	↑↑	↑↑	↑					
		High ECM Density	C	↑↑	↑↑	↑	Yes	Yes	Collective	Liquid → Solid	
			P	↓	↓	↓					

Measured trends and observations are summarized relative to the early stage MCF-10A micro-spheroid. The two spheroid systems were labeled as “Micro” and “Macro” based on the approximate number of cells contained in the multicellular clusters. “Early Stage” and “Late Stage” conditions correspond to the early (day 3–5) and late (day 7–10) stages of evolution for MCF-10A micro-spheroid. “Low ECM Density” and “High ECM Density” correspond, respectively, to 2 and 4 mg/mL collagen concentration in which macro-spheroids were embedded. Regions within spheroids were roughly separated into core (“c”) and periphery (“p”) to compare trends in the measured values (Shape Index, Motility, Cell Volume). Such trends and the associated radial gradients underlie the observation of different material phases and invasive modalities.

The column “Reference” specifies the figure where these observations are reported and discussed in detail in the following sections:

a In the MCF-10A micro-spheroid, the core approaches a jammed, solid-like phase;

b In the MCF-10A macro-spheroid, the periphery invades as a locally unjammed fluid-like phase;

c In MDA-MB-231 macro-spheroids, the invasive phenotype switches abruptly as a function of ECM density.

d We note that within MCF-10A macro-spheroids in 4 mg/mL collagen, there was a radial increase in cell volume but not SI and motility.

### Mapping a hypothetical jamming phase diagram

Tumor-ECM mechanical interactions are controlled by a variety of factors, including but not limited to fiber composition, connectivity, stiffness, porosity, nematic alignment, cell-matrix adhesion, matrix proteolysis, cellular and nuclear stiffness, contraction, and matrix deformation (Friedl et al., 2011; Wolf et al., 2013; Kopanska et al., 2016; Lautscham et al., 2015; Steinwachs et al., 2016; Lee et al., 2018; Condor et al., 2019). Among the most primitive of these interactions is mutual volume exclusion, where cells, or cells and ECM fibers, cannot occupy the same space at the same time. This physical limitation is related to geometric confinement and steric hindrance, wherein motion of self-propelled cells can be constrained by geometry (Wolf et al., 2013; Haeger et al., 2014; Mongera et al., 2018). Rather than exhaustively capturing all the aforementioned interactions, our results indicate that cell motility (Figures 2 and 3) and ECM fiber density (Figure 4) represent key differences between the various cell types and collagen concentrations used in our experiments. For this reason, we adopted a minimalist approach to ask if we can recapitulate the observed migratory phenotypes by varying two key biophysical factors: cellular propulsion and ECM density. To answer this question, we developed the minimal 2D model that characterizes in-plane cell and ECM interactions and captures the behavior of a dense cellular collective comprising the early tumor and its invasion into a dense, but porous, ECM.

**Figure 5:**
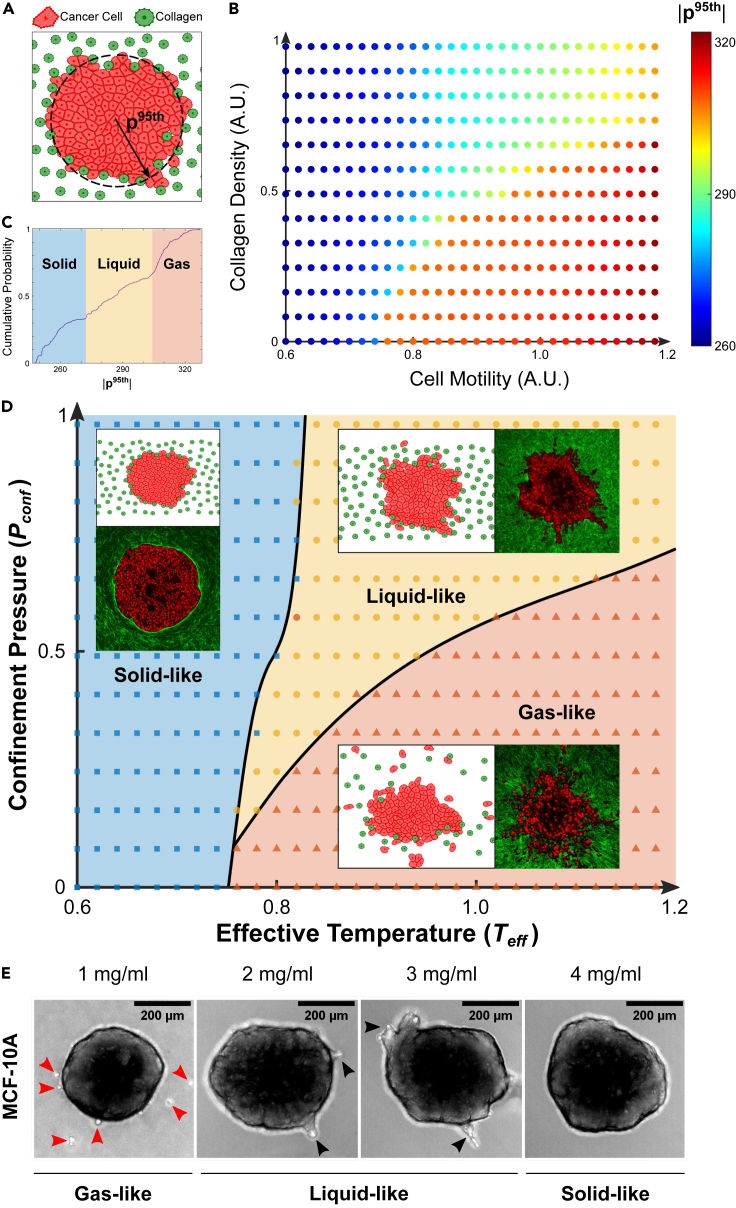
A 2D computational model of a multicellular cluster in collagen reveals that tumor invasion phenotypes and associated material states are governed by a jamming phase diagram (A) The hybrid computational model of tumor invasion into ECM is characterized by cancer cells (orange particles) that can move in random directions with varying degrees of self-propulsion (STAR Methods). At the beginning of each simulation, cancer cells are organized to form a circular collective that is surrounded by collagen (green particles), arranged randomly and with varying spatial densities (STAR Methods). At the end of each simulation, the 95^th^ percentile of the radial cell positions (**p**^95th^) with respect to the centroid of the collective is used as a readout of the degree of invasion. (B) A diagram is generated by gradually incrementing two state variables: cell motility and collagen density, both expressed in arbitrary units (A.U.). Data points are color-coded according to the mean value of **p**^95th^ over n = 10 simulations, each corresponding to randomly assigned positions of the collagen particles and orientations of the cell motility vectors. Three notable regions can be distinguished in the diagram and qualitatively correspond to solid-, liquid-, and gas-like behaviors at the invasive front (Videos S2, S3, and S4). (C) These three regions can be distinguished from distinct elbow regions in the cumulative probability distribution of **p**^95th^ generated from all simulations. We identified the 34^th^ and 64^th^ percentiles as robust thresholds (cf. Table S2) to separate solid-from liquid-like and liquid-from gas-like phases, respectively. (D) The resultant map represents a jamming phase diagram, now color-coded to indicate solid-like (blue squares), fluid-like (yellow circles), and gas-like (orange triangles) material phases. In analogy with equilibrium thermodynamic systems, here cell motility is replaced with an effective temperature (*T*_*eff*_, Box 1) while collagen density is replaced with a confinement pressure (*P*_*conf*_, Box 2). By tuning only two state variables, the model recapitulates much of the experimentally observed behaviors. For each material phase on the diagram, representative multiphoton images from experiments are shown in comparison to representative computational snapshots (insets). In the solid-like phase (blue area), a non-invasive MCF-10A spheroid in high collagen density (4 mg/mL) is shown in comparison to the result of a simulation parameterized with low cell motility (0.2) and high collagen density (0.82). In the fluid-like phase (yellow area), an MDA-MB-231 spheroid collectively invading in high collagen density (4 mg/mL) is shown in comparison to the result of a simulation parameterized with high cell motility (1.0) and high collagen density (0.82). Finally, in the gas-like phase (orange area), an MDA-MB-231 spheroid scattering into single cells in low collagen density (2 mg/mL) is shown in comparison to the result of a simulation parameterized with high cell motility (1.0) and low collagen density (0.21). Overall, we observe that at low cell motility, and thus low *T*_*eff*_, the system is homogeneously “cold” and the spheroid shows a non-invasive, solid-like behavior regardless of collagen density. However, at higher *T*_*eff*_ the collagen density, and hence *P*_*conf*_, determines fluid-like or gas-like behaviors. Phase boundaries (black lines) on the jamming phase diagram are obtained as best-fit curves that separate data points belonging to different material phases. Unlike traditional thermodynamic phase transitions, where the boundary lines mark clear transitions between material phases, in our cellular systems material transitions are continuous and smeared. Thus, the boundary lines mark regions of coexistent phases, where near each phase boundary, the material phases become indistinguishable. The proposed diagram also predicts the existence of a “triple point” where solid-, liquid-, and gas-like phases coexist, and below which direct solid-to-gas transitions occur. (E) To test the plausibility of such prediction we ran an invasion assay in graded collagen concentrations (1–4 mg/mL) using MCF-10A spheroids. which, according to the phase diagram, are characterized by a lower *T*_*eff*_ with respect to their MDA-MB-231 counterparts. The periphery of MCF-10A spheroids was found to remain solid-like and non-invasive in 4 mg/mL, to fluidize and invade collectively in 3 and 2 mg/mL (black arrowheads indicate collective protrusions) and, more importantly, to separate directly into individual gas-like cells in 1 mg/mL (red arrowheads indicate individualized cells). These findings support the direct individualization of cancer cells from a nearly jammed tumor as predicted by our jamming phase diagram.

In the model, cortical tension and cell elasticity were incorporated much as in traditional vertex models (Park et al., 2015; Bi et al., 2016). However, we modified those previous models through combination with an agent-based approach (Boromand et al., 2018) that takes into account physical interaction between cells and the ECM, as well as the possibility of single cell detachments. Each cell was assigned an elastic response to departures from a preferred area and a viscoelastic response to departures from a preferred perimeter (STAR Methods). In addition, each cell was endowed with self-propulsion of magnitude v_0,_ which acts as a vector with randomly generated polarity (Atia et al., 2018). Adjacent cells were given their own cell boundaries that moved along with the cell to which they belonged. To highlight the roles of steric hindrance and system geometry, ECM density was modeled by tuning the spatial density of matrix fibers, which are represented as randomly distributed discrete posts that are fixed in space and do not adhere to cells. Despite these simplifications, our 2D hybrid model shows a remarkably rich repertoire of dynamical behaviors and captures well the striking phenomena reported in the experimental models (Figure 5).

For the sake of simplicity, we assessed the degree of invasion at the end of each simulation based on the 95^th^ percentile of the radial cell position (**p**^95th^) with respect to the cluster centroid (Figure 5A). By modeling the behavior of cell clusters while varying both cell motility and collagen density, we found that the resulting map of **p**^95th^ (Figure 5B) depicts three notable regions, each corresponding to a distinct invasion phenotype (Figures 5C and 5D). Specifically, low values of **p**^95th^ correspond to a low degree of invasion (blue area in Figures 5C and 5D, Video S2), intermediate values of **p**^95th^ are associated with collective cell invasion (yellow area in Figures 5C and 5D, Video S3), while high values of **p**^95th^ are associated with single cell invasion (orange area in Figures 5C and 5D, Video S4). Using the cumulative probability distribution of **p**^95th^, we mapped these three regions onto a jamming phase diagram (Figures 5C and 5D;Table S2). Within this phase diagram solid-like, fluid-like, and gas-like phases dominate the invasion of the simulated spheroid in a manner that is remarkably similar to the invasive phenotypes observed experimentally (Figure 5D).

Video S2. Representative simulation showing a cell collective exhibiting a solid-like non-invasive phase, related to Figure 5 Although cells remain migratory within the collective, the cell collective does not invade into the surrounding ECM. Cells are shown as orange particles, while green particles represent the surrounding ECM. This simulation corresponds to high collagen density (0.82) and low cell motility (0.2).

Video S3. Representative simulation showing a cell collective exhibiting a liquid-like invasive phase, related to Figure 5 Cells slowly form streams that invade collectively into the surrounding ECM. Cells are shown as orange particles, while green particles represent the surrounding ECM. This simulation corresponds to high collagen density (0.82) and high cell motility (1.0).

Video S4. Representative simulation showing a cell collective exhibiting a gas-like invasive phase, related to Figure 5 Cells from the collective invade into the ECM either as single cells or as small cell clusters. Cells are shown as orange particles, while green particles represent the surrounding ECM. This simulation corresponds to low collagen density (0.21) and high cell motility (1.0).

This computational model not only recapitulated our experimental observations, but further suggested that a cancer cell collective can invade into the surrounding ECM by following a variety of trajectories within the proposed jamming phase diagram. When cell motility (v_0_) is small the cellular collective shows solid-like behavior with little migration or invasion, and thus resembles nearly jammed MCF-10A spheroids in high density collagen (Figure 5D). When v_0_ is progressively increased while keeping collagen density fixed and high, a fluid-like cellular collective flows in branches that protrude from the continuous tumor mass, much like the MDA-MB-231 spheroids undergoing a jamming transition in high collagen density (Figure 5D). When v_0_ is held fixed and collagen density is decreased, single cells and cell clusters detach from the collective, thus mimicking the gas-like dispersion observed in MDA-MB-231 spheroids that progressively re-jam in high collagen density (Figure 5D). Furthermore, when both v_0_ and collagen density are low the phase diagram predicts a “triple point” at which solid-like, fluid-like, and gas-like phases coexist. This physical picture implies a novel invasion modality, namely, a solid-to-gas transition in which cancer cells detach from the nearly jammed spheroid and individualize. We tested the plausibility of such prediction from the phase diagram by performing an invasion assay using MCF-10A spheroids in graded collagen concentrations (1–4 mg/mL). Over 48 h in 4 mg/mL collagen, MCF-10A spheroids remained solid-like. As extracellular confinement progressively reduced until collagen density reached 1 mg/mL, we observed direct detachment of single MCF-10A cells from the main spheroid and transient dissemination (Nguyen-Ngoc et al., 2012) as gas-like particles (Figure 5E and Video S5). We suggest that the numerous variables that govern collective behavior may combine so as to reduce to only two overriding variables, namely, an effective temperature (Box 1) and an effective confinement pressure (Box 2). Overall, we conclude that the proposed jamming phase diagram provides a useful guide for thought and, potentially, a unifying mechanistic interpretation of jamming/unjamming transitions in cancer invasion.

Box 1 **Effective temperature**

The standard definition of temperature is well understood, but it is sometimes useful to define an ‘effective temperature’ that has a functional equivalence. For example, Edwards and Oakeshott (1989), in a conceptual leap considered the physics governing a powder, which is representative of a wider class of an inanimate inert collective systems that includes sand piles, pastes, colloid suspensions, emulsions, foams, or slurries (Sollich, 1998). In such a collective system, thermal fluctuations are insufficient to drive microstructural rearrangements. As a result, the system tends to become trapped far from thermodynamic equilibrium (Sollich, 1998). Edwards and Oakeshott suggested that this class of collective systems might be understood in terms of the statistical mechanics of what have come to be called jammed packing. Their conjecture was as follows: of the great many possible jammed packings into which such a collective system might become trapped, in the vicinity of a jamming transition all packings become equally likely. The Edwards conjecture was validated only recently (Martiniani et al., 2017). In these inanimate inert systems, the place of energy, *E*, in thermal systems is then taken by local available volume, *V*. This assertion leads to the definition of an effective temperature, *T*_*eff*_, based upon the statistics of volume variation in jammed packings. Specifically, if in thermal systems,

T=1/(∂S/∂E)dE=TdS

then in these granular athermal systems,

$$T_{eff}=1/\left(\partial S/\partial V\right)\;dV=T_{eff}\;dS$$

where *S* is the configurational entropy.

These notions of jammed packings, configurational entropy, and an effective temperature were subsequently extended to the living epithelial monolayer by Atia et al.(2018). They showed that just as volume variation follows a *k-gamma* distribution and maximizes configurational entropy in the jammed collective inanimate system (Aste and Di Matteo, 2008; Edwards and Oakeshott, 1989), so too does cell shape variation in the jammed confluent cellular system, both *in vitro* and *in vivo* (Atia et al., 2018).

*T*_*eff*_in tumor invasion dynamics: Using hard spheres in solution as a model system for jamming, one axis of the jamming phase diagram is typically given by *k*_*B*_*T/U* where *k*_*B*_ is the Boltzmann constant, *T* is the thermodynamic temperature, and *U* in interparticle attractive energy. This ratio is akin to an effective temperature in these systems. When temperature is higher or the particles are less attractive, the system tends to be less jammed and more fluid like. In our computational model system for tumor dynamics (Figure 5), we find a similar balance between cellular propulsion and cell adhesion. If adhesion is kept constant as propulsion increases, we find that the cellular collective fluidizes. Indeed, MDA-MB-231 cells are both less adhesive (smaller effective interparticle energy) and more propulsive (higher effective temperature) than MCF-10A cells (The PS-OC Network et al., 2013). In concert with that expectation, MDA-MB-231 cells fluidize in the same surrounding matrix more easily than do MCF-10A cells (Figures 2 and 3).

Box 2 **Confinement pressure**

A variety of physical forces act to confine and direct collective cellular behavior. For example, cells within the tumor experience compressive stress due to uncontrolled growth. Indeed, Jain and colleagues have shown that the tumor interior develops compressive stresses large enough to collapse the intra-tumor vasculature (Tse et al., 2012; Stylianopoulos et al., 2013; Nia et al., 2020). Literature developed by us (Zhou et al., 2009; Guo et al., 2017; Han et al., 2020; De Marzio et al., 2021) and others (Finan et al., 2009; Khavari and Ehrlicher, 2019) shows that these compressive stresses can lead to systematic decreases of cell and nuclear volumes with increasing compressive stress. Additionally, within multicellular tumor models, and within the human tumor explants, nuclear volume varies appreciably and systematically both in space and time (Han et al., 2020). As such, changes in cell and nuclear volume are sensitive to the local microenvironment (Discher et al., 2009; Swift et al., 2013; Guo et al., 2017), although whether or not they can serve as a remote pressure sensor, as some suggest (Khavari and Ehrlicher, 2019), remains debatable. It is notable that across these different systems, cell volume appears to change in close accordance with the well-known Boyle-Van’t Hoff relationship (Finan et al., 2009; Zhou et al., 2009; Guo et al., 2017; Khavari and Ehrlicher, 2019). These volume changes also appear to be associated with changing mechanical properties of the cell, with a strong increase in cell stiffness as cell volume decreases. From the Boyle-Van’t Hoff relationship, calculation of the bulk osmotic modulus, *B*, is straightforward (Zhou et al., 2009):

$$B=-V\;\left(\partial \Pi /\partial V\right)=Nk_{B}TV/{\left(V-V_{\min}\right)}^{2}$$

where *Π* is the osmotic pressure, *N* is the total number of osmolytes, *k*_*B*_ is the Boltzmann constant, *T* is the temperature, *V* is the volume, and *V*_*min*_ is the osmotically inactive volume.

For reasons that remain unclear, both cortical and cytoplasmic stiffness are orders of magnitude smaller but follow the same functional trend (Zhou et al., 2009; Guo et al., 2017). As is described below, increasing cell stiffness may influence jamming behavior and as such understanding these forces remains critical.

*P*_*conf*_in tumor invasion dynamics: In jamming behavior of colloids, micro-gels or many collective systems, both particle number density and particle stiffness play critical roles; higher number densities and stiffer particles tend to promote jamming (Liu and Nagel, 1998).When these factors are held constant but collagen density is high, our computational model shows that the cellular collective can be either solid-like or fluid-like depending on propulsion (and therefore *T*_*eff*_; Box 1) but cell escape as a gas is not possible (Figure 5). However, when collagen density is lowered, the cellular collective can become gas-like (depending on *T*_*eff*_), in which case cells escape readily. Changing collagen density in this computational model is akin to changing an effective confinement pressure, *P*_*conf*_, in which case the matrix is imagined to comprise a vessel that acts to confine the jammed collective. In concert with these notions, experiments show that MDA-MB-231 cells are softer and exert greater propulsive forces than do MCF-10A cells (The PS-OC Network et al., 2013) and, as expected, escape more easily into an equivalent matrix (Figures 3 and 4).

Video S5. DIC time-lapse movies recorded over the course of 48 h for MCF-10A spheroids embedded in graded concentrations of collagen (1–4 mg/mL), related to Figure 5 The periphery of MCF-10A spheroid remained solid-like and non-invasive in 4 mg/mL, formed collective protrusions as collagen concentration was progressively reduced to 3 and 2 mg/mL. Notably, as collagen concentration was further reduced to 1 mg/mL, single cell detachment was observed while the main spheroid remained solid-like.

## Discussion

The principle finding of this report is that cell morphology, packing, migration, and invasion, as well as their changes in space and time, are governed in large part by non-equilibrium phase separation. The resulting phases include a jammed solid-like phase, and unjammed fluid-like and gas-like phases. Depending on cell and matrix properties, the tumor mass can invade collectively either by undergoing unjamming, as in the case of MCF-10A spheroids in 2 mg/mL collagen, or by means of progressive re-jamming and confinement, as in the case of MDA-MB-231 spheroids in 4 mg/mL collagen. These diverse phenotypes are unified by a novel jamming phase diagram (Figure 5D). Despite its apparent simplicity, this phase diagram leads to a novel physical picture of collective invasion into ECM and the roles of cell jamming and unjamming. First, this phase diagram—derived from a computational model but supported by experimental observations—points to the observed phase separation as being governed mainly by collagen density and cell motility, which are factors that have been considered previously (Haeger et al., 2014; Bi et al., 2016) but not in this context. Second, in addition to the previously identified modes of fluid-like invasion upon deregulation of adherens junctions (Ilina et al., 2020), we find a third mode of collective invasion. In this mode, ECM confinement progressively jam/solidify highly motile cells which invade collectively as a solid-like flock. Together, this picture suggests that tumor invasion involves diverse routes toward phase separation. As a demonstration of one such route, the phase diagram also predicts a distinctive and novel solid-to-gas transition, reminiscent of sublimation, which we confirmed experimentally (Figure 5E). This novel jamming phase diagram, in turn, leads to the suggestion that many of the numerous factors that determine tumor cell migration, packing shape and invasiveness may map into a much smaller set of ‘effective’ thermodynamic variables, such as an effective temperature (Box 1) and an effective confinement pressure (Box 2).

As used here, the phrase ‘coexistence’ of material phases has two distinct but interrelated connotations. As suggested previously, in the vicinity of a phase boundary seemingly modest changes of cellular or ECM properties may have the potential to precipitate striking changes of material phase and invasion phenotype (Fredberg, 2014). The second connotation suggests that in the same spheroid the cellular collective can express macroscopic regional differences, such as a solid-like core coexisting with a fluid-like invasive branch. In MCF-10A spheroids, for example, cells at the periphery compared with cells near the core tend to be systematically more elongated, more variable in shape, and more migratory (Figures 1 and 2). Both connotations are indicative of glass-like dynamics and highlight the non-equilibrium nature of the observed phase transitions, even though the proposed phase diagram (Figure 5D) resembles that from equilibrium thermodynamics. In the context of active force fluctuations and associated metabolism, cells at the periphery are also more dynamic (Guo et al., 2014; Han et al., 2020). Conversely, for post-metastatic MDA-MB-231 spheroids, cells at the core are larger, more variable in shape, and more migratory with respect to their MCF-10A counterparts, while the periphery is highly sensitive to changes in collagen density (Figures 3 and 4). Compared to MCF-10A cells, they also generate higher traction forces (The PS-OC Network et al., 2013) and are more metabolically responsive (Mah et al., 2018). Together, this constellation of structural, migratory, mechanical, and metabolic factors is consistent with the existence of an effective temperature, *T*_*eff*_, that is spatially heterogeneous. Such a physical picture would help to explain, and perhaps to generalize, the x-axis of the hypothesized jamming coexistence phase diagram (Figure 5D; Box 1). As regards the y-axis of the hypothesized phase diagram, it is well established that both solid and fluid stresses within the spheroid core are compressive, and that cellular and nuclear volumes in the core are reduced, as if under compression (Stylianopoulos et al., 2013; Han et al., 2020). Such a compressive state of stress is thought to arise in part from cellular proliferation under the constraint to ECM confinement (Helmlinger et al., 1997). In addition, osmotic pressure decreases cellular volume, increases cell stiffness and thereby decreases invasiveness of peripheral cells (Han et al., 2020). It is becoming increasingly clear that physical cues from the ECM can cause spatially heterogeneous migratory modes in initially homogeneous cell populations. Recent work using synthetic hydrogels shows that fiber density and bulk stiffness distinctively contribute to the migratory switch observed spheroid invasion (Hiraki et al., 2021). However, in natural hydrogels, such as collagen, fiber density and stiffness are inexorably related, and our own results show that increasing fiber density stiffens the ECM (Figures 4 and S8), thus potentially increasing the solid stress acting on proliferating spheroids. Stiffening of collagen fibers via non-enzymatic glycation also causes structural differences, with glycated networks displaying lower fiber density and larger pore diameters with respect to non-glycated controls (Hall et al., 2016). Hence, glycation can shift the critical collagen density at which the transition between single cell and collective invasion occurs. While in our work the critical collagen concentration lies between 2 and 3 mg/mL, glycation is likely to increase that value as suggested by data from Suh et al. (2019) where a higher number of invading single cells is detected in glycated with respect to control collagen at a fixed concentration of 3.5 mg/mL. It remains unclear, however, how fiber density and stiffness, solid stress due to compression, shear, or tension from neighboring cells and the ECM, interstitial fluid stress, and osmotic stress combine to generate the hypothesized confinement pressure *P*_*conf*_. Such connections, if they could be established, would help to explain, and perhaps to generalize, the y-axis of the hypothesized jamming coexistence phase diagram (Figure 5D; Box 2).

A central role of the EMT in tumor cell motility, invasiveness, and metastasis, is well established but has recently become a point of contention (Brabletz et al., 2018). A recent report observed various modes of collective migration in unjammed cancer cell collectives displaying a range of EMT status upon down-regulation of E-cadherin (Ilina et al., 2020). Most notably, by tuning cell-cell adhesion strength different fluid phases were identified in triple-negative 4T1 breast cancer cells migrating collectively along a 2D collagen-glass interface. Highly motile and elongated cancer cells can, in fact, migrate with a high degree of coordination in the presence of strong cell adhesions—an active nematic phase—or with a low degree of coordination in the presence of weak cell adhesions, an active fluid phase (cf. Extended Data Figure 7 in Ilina et al., 2020). Contrary to that report, our data from triple-negative MDA-MB-231 breast cancer cells invading collectively into high density collagen display a consistent radial trend toward reduced cell volumes, more regular shapes, and slower motions (Figure 3) suggestive of proximity to a more jammed state. The slower kinetics of the resulting invasive branches is characterized by a reduced tangential velocity and coordinated radial motions between neighboring cells at the invasive periphery (Figures 3, 4, and S7D). This seemingly paradoxical observation of collective invasion by jamming is consistent with the proposed existence of a migrating ‘solid-flock’ (Giavazzi et al., 2018), where cells are internally rigid in a solid-like state with no local rearrangement, yet can exhibit collectively directed motion due to suppression of motility fluctuations transverse to the mean migration direction. A possibility is that collective invasion in mesenchymal cells is due to the development of supracellular actin cables (Grosser et al., 2021) which could increase interfacial surface tension and coordinate motions in higher density ECM. While we did not observe such actin structures in MDA-MB-231 spheroids (Figure S9), other mechanisms, including cell-ECM signaling and cell contractility (Lee et al., 2021), are likely to play a key role. Taken together, our observations indicate that both jamming and unjamming are highly influential in determining tissue fluidity and collective invasion patterns. Therefore, in addition to a linear path toward progressive tissue fluidization via unjamming (Palamidessi et al., 2019; Ilina et al., 2020), ECM confinement may restore ordered migratory invasion via progressive re-jamming and promote next-neighbor coordination in weakly adhesive cells. The exact mechanisms remain ill-defined, but our findings reveal that jamming and unjamming transitions represent a much richer process than previously anticipated, while illustrating the usefulness of a unified interpretation through a jamming phase diagram

Compared to inert materials, cellular collectives are biologically active and displaced far from thermodynamic equilibrium. Thus, the collective material phases identified here, and the associated jamming/unjamming transitions, are not to be confused with first order or second order transitions occurring in systems close to thermodynamic equilibrium. Like the glass transition (Berthier et al., 2019), jamming/unjamming transitions generally display a discontinuity in the number of contacts, which are characteristic of first-order phase transitions, but display diverging correlation length scales, which are characteristic of second-order phase transitions (O'Hern et al., 2002; Park et al., 2015; Cubuk et al., 2017). Similarly, we find that cell migration shows smooth radial changes in invading tumor spheroids (Figures 1, 2, and 3) but sharp transitions as a function of collagen concentration (Figure 4). As opposed to a phase transition that is binary and sharp, as might occur in an equilibrium system, the transition between a jammed and an unjammed cellular phase is continuous and smeared both in space and in time (Angelini et al., 2011; Mitchel et al., 2020). Just as there is no latent heat and no structural signature of melting for inert materials approaching the glass melting point, so too in invading tumor spheroids there is no sharp transition in cellular shapes or migration speeds. Therefore, the material phase of a cellular collective needs to be defined via functional terms – cellular migratory persistence, cooperativity, a target shape index, and cellular migratory propulsion (Park et al., 2015; Bi et al., 2015, 2016).Yet, despite these differences, the cell collectives herein are observed to transit various material states within a phase diagram that bear superficial resemblance to that of common thermodynamic systems. These results suggest that collective cellular migration, invasion, and escape from a cellular mass involve biophysical processes far richer than previously anticipated, but may be governed by basic physical principles. We have shown how specific cell and ECM properties can be reduced to a set of ‘effective’ thermodynamic variables describing the material phase of the invasive cell collective, and thus mapping a jamming phase diagram of tumor invasion. How the local material phase of the cellular collective and its mechanical properties might impact the emergence of driver mutations remains unknown. Deformation of the cell and its nucleus associated with migration within a highly confining microenvironment is known to cause loss of nuclear envelope integrity, herniation of chromatin across the nuclear envelope and DNA damage (Denais et al., 2016), but the impact of cell and nuclear elongation in connection with unjamming remains unstudied. Conversely, how driver mutations and resulting subclonal heterogeneities might impact the local material phase is also unclear. When such interactions become appreciable, tumor dynamics would then be seen to be a multifaceted problem in mechanogenetics (Pfeifer et al., 2017).

### Limitations of the study

Experiments reported here were performed using two cell lines, one that expresses predominantly epithelial characteristics (MCF-10A) while the other expresses primarily mesenchymal characteristics (MDA-MB-231). Our experimental observations highlight that cells in the core of MDA-MB-231 spheroids are more motile than cells from MCF-10A spheroids regardless of experimental conditions, consistent with enriched expression in migratory genes in MDA-MB-231 cells (Lehmann et al., 2011). Even though our experiments did not vary cell motility in a controlled fashion, our hybrid computational model systematically controlled for cell motility as well as ECM density, thus being able to recapitulate the experimentally observed invasion patterns. Due to limitations of the *in vitro* system adopted herein, clinical implications of the proposed jamming phase diagram require further investigation. Inherent limitations are associated with both imaging and segmentation of single cells within large multicellular collectives. For this reason, we developed an alternative approach to estimate cell shape. First, we accurately identified the spatial locations of cell nuclei within a spheroid, which were then used as seeds for Voronoi-based tiling that approximated individual cell shapes within the contiguous cell mass; such an approach yielded a linear trend between Voronoi and nuclear volumes consistent with the linear trends reported using membrane stains (Guo et al., 2017; Han et al., 2020). Furthermore, our approximation of cell shape within spheroids is consistent with theoretical work identifying the critical shape index for 3D phase transitions (Merkel and Manning, 2018). Our hybrid computational model identifies cell motility and ECM density as the main contributors toward the hypothesized effective temperature and confinement pressure, but it remains to be elucidated whether, and how, these effective thermodynamic variables are impacted by cellular and nuclear stiffness, cell adhesion, cortical tension, actomyosin contractility, proteolytic activity, as well as ECM stiffness and alignment.

## STAR★Methods

### Key resources table

**Table:** 

REAGENT or RESOURCE	SOURCE	IDENTIFIER
**Antibodies**		

GAPDH (D16H11) Rabbit mab	Cell Signaling Technology	Cat. #5174; RRID:AB_10622025
Vimentin (D21H3) Rabbit mab	Cell Signaling Technology	Cat. #5741; RRID:AB_10695459
E-Cadherin (24E10) Rabbit mab	Cell Signaling Technology	Cat. #3195; RRID:AB_2291471
N-Cadherin (D4R1H) Rabbit mab	Cell Signaling Technology	Cat. #13116; RRID:AB_2687616
Goat anti-Rabbit IgG cross-adsorbed secondary Ab, Alexa Fluor 488	Thermo Fisher Scientific	Cat. #A-11008; RRID:AB_143165
Goat anti-Rabbit IgG cross-adsorbed secondary Ab, Alexa Fluor 594	Thermo Fischer Scientific	Cat. #A-11012; RRID:AB_2534079
Alexa Fluor 568 Phalloidin	Thermo Fischer Scientific	Cat. #A12380

**Chemicals, peptides, and recombinant proteins**		

DMEM/F-12	Thermo Fisher Scientific	No. 11330032
Horse Serum	Thermo Fisher Scientific	No. 16050122
EGF	Thermo Fisher Scientific	No. 10605HNAE
Hydrocortisone	Sigma-Aldrich	No. H0888
Cholera toxin	Sigma-Aldrich	C8052
Matrigel	Corning	No. 354234
Collagen I	Corning	No. 354249
Quadrol	Sigma-Aldrich	No. 122262
Triton X-100	Sigma-Aldrich	No. T8787
Urea	Fisher Scientific	No. U15
Paraformaldehyde	Fisher Scientific	No. AAJ19943K2
DAPI	Fisher Scientific	No. D1306

**Experimental models: Cell lines**		

MCF-10A	ATCC	CRL-10317
MDA-MB-231	ATCC	CRM-HTB-26

**Software and algorithms**		

ImageJ v.1.52g	National Institute of Health	https://imagej.nih.gov/ij/download.html
Matlab R2019a	Mathworks	https://www.mathworks.com/login?uri=%2Fdownloads%2Fweb_downloads%2Fdownload_release%3Frelease%3DR2019a
CT-FIRE	(Bredfeldt et al., 2014)	http://loci.wisc.edu/software/ctfire
Agent-based model	Zenodo	https://doi.org/10.5281/zenodo.5542509

### Resource availability

#### Lead contact

Further information and requests for resources and reagents should be directed to and will be fulfilled by the lead contact, Jeffrey J. Fredberg (jjf@harvard.edu).

#### Materials availability

This study did not generate new unique reagents.

### Experimental model and subject details

#### Cell lines and culture media

Non-tumorigenic MCF-10A and metastatic MDA-MB-231 breast epithelial cell lines were purchased from American Type Cell Culture Collection (ATCC) and cultured using standardized media and conditions (Kim et al., 2020; Han et al., 2020). MCF-10A cells were cultured in DMEM/F-12 (ThermoFisher, No. 11330032) supplemented with 5% horse serum (Invitrogen, No. 16050122), 20 ng/ml EGF (Peprotech, AF10015; ThermoFisher, No. 10605HNAE), 0.5 mg/ml hydrocortisone (Sigma-Aldrich, No. H0888), 100 ng/ml cholera toxin (Sigma-Aldrich, C8052), 10 μg/ml insulin (Sigma-Aldrich, No. I1882). MDA-MB-231 cells were culture in DMEM (Corning, No. 10013CV), supplemented with 10% fetal bovine serum (ATCC, No. 302020). Both media recipes contained 1% penicillin/streptomycin (ATCC, No. 302300; ThermoFisher, No. 15140122). Cells were maintained at 37°C and 5% CO_2_ in a cell culture incubator.

### Method details

#### Spheroid formation

Two distinct protocols were used to generate the micro- and macro-spheroid models used in this study. First, micro-spheroids were formed by trypsinizing and embedding MCF-10A cells within an interpenetrating network (IPN) consisting of 5 mg/ml Alginate (FMC Biopolymer) and 4 mg/ml Matrigel (Corning, No. 354234) as previously shown (Chaudhuri et al., 2014; Han et al., 2020). Cells were mixed with the gel precursor solution, which was allowed to gel inside an incubator before adding culture media. The shear modulus of the double network can be tuned via calcium cross-linking and was herein set to 300 Pa to reproduce the stiffness of malignant breast tissue (Chaudhuri et al., 2014). Within the IPN, cells proliferated to form micro-spheroids that began invading into the gel after approximately 7 to 10 days in culture. Second, MCF-10A and MDA-MB-231 macro-spheroids were generated by seeding approximately 10^3^ cells in each of the 96 wells of an ultra-low attachment plate (Corning, No. 07201680) and allowed to form for 48 h in the presence of 2.5% v/v Matrigel. We verified that addition of a small volume fraction of Matrigel allows the formation of MDA-MB-231 spheroids, which would otherwise form only loose aggregates (Figure S6) (Ivascu and Kubbies, 2006). MCF-10A spheroids were formed under the same conditions to ensure consistency. Once formed, individual spheroids surrounded by a small volume of media were transferred in microwells (10 mm in diameter) inside glass bottom 6-well plates (MatTek, No. P06G-0-10-F) by pipetting 5 μl drops on each of the coverslips. Each spheroid was covered by 145 μl of ice-cold, rat-tail collagen I solution to achieve a total volume of 150 μl and a specific collagen concentration in each microwell. Collagen solutions were prepared by mixing acid-solubilized collagen I (Corning, No. 354249) with equal volumes of a neutralizing solution (100 mM HEPES buffer in 2x PBS) (Harjanto et al., 2011). The desired collagen concentration was reached by adding adequate volumes of 1x PBS. Collagen solutions at different concentrations (1, 2, 3, and 4 mg/ml) polymerized for 1 h at 37°C. The cell culture plates were rotated every minute for the first 10 min of polymerization to guarantee full embedding of the spheroid within the 3D collagen matrix. Finally, 2 ml of culture media was added and the 3D organotypic culture was placed inside the incubator for a variable amount of time. Media was refreshed every two days.

#### Multiphoton microscopy

In order to image cell nuclei within large spheroids, we adapted a technique known as CUBIC (Clear, Unobstructed Brain/Body Imaging Cocktails) which was originally developed to enable optical clearing and high-resolution imaging of murine organs (Susaki et al., 2015). Briefly, CUBIC employs hydrophilic reagents to remove lipids (the main source of scattering within tissues), while preserving fluorescent proteins. Following the original protocol by Susaki et al.(2015), we prepared a mixture of 25% wt urea (Fisher Scientific, No. U15), 25% wt Quadrol (N,N,N′,N'-Tetrakis(2-hydroxypropyl)ethylenediamine, Sigma-Aldrich, No. 122262), 15% Triton X-100 (Sigma-Aldrich, No. T8787), and dH_2_O. At regular intervals after embedding, spheroids were fixed overnight using cold 4% PFA (Fisher Scientific, No. AAJ19943K2), and washed out twice using 1x PBS. Samples were pretreated for 2 h in 2 ml of ½ diluted CUBIC reagent (50% vol/vol dH_2_O) and then immersed in 1 ml CUBIC reagent with 2 μM DAPI (Fisher Scientific, No. D1306) under gentle shaking at room temperature. The CUBIC reagent and DAPI were refreshed every two days and samples were cleared for up to 14 days prior to imaging. The 3D organization of optically cleared spheroids in collagen was imaged using a Bruker Ultima Investigator multiphoton microscope (MPM). The laser beam was focused onto the spheroids through a 16× water-immersion objective (Nikon, 0.8 N.A., 3 mm working distance) mounted in upright configuration. We used an excitation wavelength of 880 nm to image both invading spheroids and collagen. Two-photon excitation fluorescence (TPEF) from DAPI stained nuclei was collected through a bandpass filter centered at 550 nm with a bandwidth of 100 nm, while SHG signal from the collagen matrix was collected through a bandpass filter centered at 440 nm with a bandwidth of 80 nm. Images were collected using 1024 ×1024 pixels at a resolution of 0.805 μm/pixel and a pixel dwell time of 10 μs. Stacks were acquired using 5 μm steps and a thickness (variable in the range 400–1000 μm) that was determined depending on the spheroid size and degree of invasion. Laser power and photomultiplier tube voltage were increased to maintain a nearly constant signal across the spheroid and interpolated linearly though the PrairieView software during acquisition.

#### Immunofluorescence

MCF-10A and MDA-MB-231 cells were cultured as a 2D monolayer on glass bottom plates for 6 days. Cells were fixed using either 4% PFA (20°C for 30 min) or Methanol (−20°C for 30 min), permeabilized with PBS/0.2% Triton X-100 (20°C for 10 min), followed by incubation with primary antibody diluted in PBS/10% goat serum/1% BSA/0.2% Triton X-100 (4°C overnight), rinsed, and incubated with secondary antibody diluted in PBS/10% goat serum/1% BSA/0.2% Triton X-100 (20°C for 1 h), and counterstained with 2nM DAPI (20°C for 10 min). Cells were rinsed and stored in 1x PBS for confocal fluorescence microscopy. Imaging was performed using an Olympus FV3000 confocal microscope equipped with a 20x/0.75N.A. objective. Image stacks from all samples are shown as maximum intensity projections. Proteins of interest were detected with the following primary antibodies from Cell Signaling Technologies: E-cadherin (1:200, #3195), Vimentin (1:100, #5741). Fluorescent labeling was carried out using the following secondary antibodies from Thermo Fisher Scientific: goat anti-rabbit AlexaFluor 488 (1:500, A-11008), goat anti-rabbit AlexaFluor 594 (1:500, A-11012). F-actin was stained using AlexaFluor 568 Phalloidin (1:400, A-12380).

#### Western blotting

Lysates from MCF-10A and MDA-MB-231 monolayers were collected into ice-cold RIPA buffer with protease inhibitor cocktail and processed by incubation on ice for 30 min with sonication halfway through. Lysates were cleared by centrifugation at 13000g for 5 min and the supernatant was collected. Lysates were mixed with equal volume Laemmli 4x sample buffer (BioRAD) with 1M DTT and boiled for 6 min. Proteins of interest were detected using standard Western blotting with the following antibodies, all from Cell Signaling Technologies: E-cadherin (1:10,000, #3195), N-cadherin (1:1000, #13116), Vimentin (1:1000, #5741), GAPDH (1:10,000, #5174).

#### Collagen structure and mechanics

Acellular collagen gels at concentrations of 1–4 mg/mL were polymerized within PDMS molds to form cylindrical plugs. After collagen self-assembly, the gels were kept hydrated via addition of 2 mL of 1x PBS.

##### Structure

The microstructural features of acellular collagen networks were characterized using previously published methods (Ferruzzi et al., 2019). Briefly, collagen gels were imaged using a Bruker Ultima Investigator MPM and a 60× oil-immersion objective. Image stacks of dimensions 78.9 μm × 78.9 μm × 10 μm (x–y–z) were acquired with 1 μm steps at a resolution of 0.076 μm/pixel. Masks that identify collagen fibers from SHG signal were generated using a global intensity threshold that was determined empirically (Ferruzzi et al., 2019; Raub et al., 2007). Collagen gels were treated as biphasic mixtures (Ateshian et al., 1997) made of two constituents: a solid (*s*, collagen) and a fluid (*f*, 1x PBS). The mixture is fully saturated, that is *V*^*s*^+*V*^*f*^=*V* or *φ*^*s*^+*φ*^*f*^=1, where *V*^*α*^ and *V* represent constituent and total volumes, *φ*^*α*^=*V*^*α*^/*V* represent volume fractions with *α* = *s*, *f*. Due to the random orientation of self-assembled collagen fibers, the volume fraction of collagen can be calculated from the respective area fraction (Dullien, 2012). Hence, we computed *φ*^*α*^=*V*^*α*^/*V*≈*A*^*α*^/*A*, here *A*^*s*^ represents the masked area occupied by collagen fibers and *A* represents the total imaged area (78.9 × 78.9 μm^2^). The fluid volume fraction – or porosity – was then calculated as *φ*^*f*^=1−*φ*^*s*^. Individual collagen fibers were identified using CT-FIRE, a validated algorithm for segmentation of microscopy images and extraction of fiber geometry and alignment (Bredfeldt et al., 2014). Examples of collagen fiber identification via CT-FIRE segmentation are shown in Figure 4D. Collagen fiber density was calculated for each slice as the number of segmented fibers divided by the sampled volume (78.9 × 78.9 × 1 μm^3^) and averaged across each image stack.

##### Mechanics

The macroscopic properties of cylindrical collagen samples polymerized within PDMS wells were estimated using a microscale compression system (Cell Scale, Waterloo, ON, Canada). Cylindrical collagen plugs with a diameter of 5mm and height of 2mm were prepared using established protocols (Ferruzzi et al., 2019) and subjected to uniaxial unconfined compression testing by means of a 6mm х 6mm platen fixed to a microbeam of known elastic modulus (411 GPa) and length (57–59 mm). The microbeam diameter Φ dictates the overall force resolution and was found to depend on collagen concentration, which led us to use the following diameters determined by means of preliminary tests: Φ = 203.2 μm (1 mg/ml), Φ = 304.8 μm (2 mg/ml), and Φ = 406.4 μm (3 and 4 mg/ml). Five stress relaxation steps were imposed by applying a deformation equal to 3% of the unloaded gel height, followed by a 180 s hold. In virtue of the nearly linear responses observed at equilibrium, the bulk mechanical behavior measured under unconfined compression was modeled using a compressible neo-Hookean free energy density function *W*

(Equation 1) $$W=\frac{c}{2}\left(\bar{I_{C}}-3\right)+\frac{1}{D}{\left(J-1\right)}^{2},$$

where $\bar{I_{C}}=J^{-2/3}trC$, *J*=*det* **F**, and **C**=**F**^*T*^**F**. **F** is the deformation gradient which, in confined compression, assumes diagonal form **F**=diag(*λ*_*r*_,*λ*_*θ*_,*λ*_*z*_) where *λ*_*z*_=*h*/*H* is the ratio between loaded (*h*) and unloaded (*H*) gel thickness – or axial stretch – imposed by the microbeam, while *λ*_*r*_=*λ*_*θ*_=*r*/*R* is the ratio between loaded (*r*) and unloaded (*R*) gel radii captured using a digital camera and measured using Image J. The parameter *D* was determined by imposing a zero radial stress (traction-free boundary condition), while the bulk shear modulus *c* was found by fitting the axial stress under the constraint *c*>0.

#### Agent-based modeling

We developed a computational model that represents a hybrid between vertex (Bi et al., 2016) and particle-based (Boromand et al., 2018) approaches. On a 2D computational domain, 152 cells were generated by a Voronoi tessellation of points randomly seeded (with Poisson sampling) in a circular area to form a cellular collective resembling the cross-section of a tumor spheroid. Individual cells within the cellular collective were endowed with a randomly oriented cell motility vector v_0_, viscoelastic mechanical properties, and cell-cell interactions. Overall, the cellular collective was modeled as a confluent tissue, with no birth and death events, and was considered homogenous (in terms of cell properties such as adhesion and inherent motility). Collagen was represented as posts that are randomly distributed around the cell collective and was modeled as non-motile and rigid, and thus confining the cells. To capture all invasion patterns observed experimentally, the model was configured with gradual increase in the magnitude of the cell motility vector v_0_ and the density of collagen particles, which were chosen as state variables, while keeping constant all other simulation parameters. To account for the random orientation of the motility vector and for the randomness in the spatial configuration of collagen particles, we ran 10 simulations (n = 10) for each pair of state variables (i.e., for each data point in Figure 5B) and used the averaged result to represent the spheroid behavior for each combination of state variables. At the end of each simulation, the mean distance of the outermost 5% cells from the cellular collective centroid—that is the 95% percentile of the radial cell positions (**p**^95th^)—was used to represent the overall invasive behavior of the cellular collective. This metric was then pooled from all simulations across the range of examined values and generated a cumulative distribution of **p**^95th^ (Figure 5C) which was used to identify the threshold criteria that best separated the various invasive phenotypes and associated material phases. Simulated spheroids with **p**^95th^ below the 34th percentile were labeled as solid-like, while between the 34th and 64th percentiles were labeled as fluid-like, and above the 64th percentile were labeled as gas-like (Figure 5D). Within the model, these different material phases indicated different spheroid behaviors: a solid-like behavior represents lack of invasion, a fluid-like behavior represents collective invasion, and a gas-like behavior represents single cell invasion. Mapping the material phases and invasion patterns for each state variable combinations resulted in the jamming phase diagram. Sensitivity analysis showed the choice of exact threshold criteria, within the examined range, did not significantly impact configuration of the resultant phase diagram (Table S2). Additionally, mean cell shape (defined as *perimeter*/$\sqrt{\mathrm{area}}$ for cells in 2D), for cells in the solid-like phase were smaller (p<0.001) than cells in the fluid-like phase. A detailed description on each component of the model and ensuing analysis is described below.

##### Cell mechanics

The cell membrane is modeled as viscoelastic, with neighboring points on the membrane interacting through both an elastic force $F_{ij}^{e}$ and a viscous force $F_{ij}^{v}$ that act along the cell perimeter (Jamali et al., 2010). These are respectively described by the following equations,

(Equation 2) $$\begin{array}{l} F_{ij}^{e}=-k_{m}\left(l_{ij}-l_{ij,0}\right)u_{ij}^{ƒ},\\ F_{ij}^{v}=\gamma_{m}v_{ij}, \end{array}$$

where *k*_*m*_ is the elastic constant for the cell membrane, *l*_*ij*_ is the current distance between adjacent points *i* and *j* on the membrane, *l*_*ij*,0_ is the respective equilibrium distance, and $u_{ij}^{ƒ}$ is the unit vector parallel to the segment *ij*, while *γ*_*m*_ is the viscosity coefficient for the cell membrane, and **v**_*ij*_ is the relative velocity of point *j* with respect to point *i*. Moreover, a bending force $F_{ij}^{b}$ is introduced to correct for any inward or outward bending of the membrane during the simulation. This force is described as follows:

(Equation 3) $$\begin{array}{l} F_{ij}^{b}=\frac{k_{b}}{l_{ij}}\left(\pi -\theta_{ijk}\right)u_{ij}^{⊥},\\ F_{jk}^{b}=\frac{k_{b}}{l_{jk}}\left(\pi -\theta_{ijk}\right)u_{jk}^{⊥}, \end{array}$$

where*k*_*b*_ is a bending stiffness of the cell membrane, *l*_*ij*_ and *l*_*jk*_ are the lengths of segments *ij* and *jk*, *θ*_*ijk*_ is the angle between segments *ij* and *jk*, and $u_{ij}^{⊥}$ and $u_{jk}^{⊥}$ are unit vectors normal to segments *ij* and *jk* pointing towards the cell cytoplasm. It should be noted that these forces are aimed to align adjacent segments to achieve a smoother cell surface.

The cell body is also modeled as viscoelastic, with two main types of forces governing its mechanical behavior. First, an area-driven force $F_{ij}^{a}$ which represents the surface tension resisting to changes in the cell area. This is described as follows:

(Equation 4) $$F_{ij}^{a}=k_{a}\left(a-a_{0}\right)u_{ij}^{⊥},$$

where *k*_*a*_ is the elastic constant for the cell body, *a* is the current cell area, *a*_0_ is the equilibrium cell area, and $u_{ij}^{⊥}$ is the unit vector normal to segment *ij*. Second, an elastic force $F_{ni}^{e}$ and a viscous force $F_{ni}^{v}$ act along the actin fibers distributed radially along the cell cytoskeleton (Jamali et al., 2010; Ujihara et al., 2011). These fibers connect the nucleus (*n*) with a given point (*i*) on the cell membrane. These are respectively described by the following equations:

(Equation 5) $$\begin{array}{l} F_{ni}^{e}=-k_{f}\left(l_{ni}-l_{ni,0}\right)u_{ni}^{∥},\\ F_{ni}^{v}=\gamma_{f}v_{ni}, \end{array}$$

where *k*_*f*_ is the elastic constant for actin fibers, *l*_*ni*_ is the current fiber length, *l*_*ni*,0_ is the equilibrium fiber length, $u_{ni}^{∥}$ is the unit vector parallel to the segment *ni*, while *γ*_*f*_ is the viscosity coefficient for actin fibers, and **v**_*ni*_ is the relative velocity of point *i* on the membrane with respect to point *n*, the nucleus.

Each cell possesses an inherent or self-propelled motility that can be tuned via the propulsion parameter *v*_0_, similar to the one described by Bi et al. (2016). Cell *n* receives a randomly generated polarity vector (*cosθ*_*n*_,*sinθ*_*n*_) along which the cell exerts a self-propulsion force with a constant magnitude *v*_0_/*μ*, where *μ* represents the inverse of the frictional drag coefficient (Bi et al., 2016). The polarity vector undergoes rotational diffusion by a Gaussian white-noise process with zero mean and variance *D*_*r*_, with *D*_*r*_ representing the rotational noise strength which is kept constant throughout the simulation.

##### Cell–cell interactions

Physical interactions between cells are modeled in the form of a Lennard-Jones potential (Narang et al., 2011). While the rejection component in the potential prevents cells from overlapping, the attraction component models cell-cell adhesive forces present in tissues. A notable example is given by the adherens junctions that are known to maintain structural stability of cell monolayers and allow functions such as cell communications and collective movements (Hartsock and Nelson, 2008). The inter-cellular force **F**_*LJ*_ arising from the Lennard-Jones potential is described as follows,

(Equation 6) $$F_{LJ}=k_{LJ}\left[{\left(\frac{r_{0}}{r}\right)}^{p}-2{\left(\frac{r_{0}}{r}\right)}^{q}\right]\frac{1}{r^{2}}u_{r}^{ƒ},$$

where*k*_*LJ*_ is the constant that characterizes the strength of the interaction, *r* is the current separation distance (minimum distance between points on the membranes of two interacting cells), *r*_0_ is the maximum separation distance for rejection, 2*r*_0_ is the maximum distance for interaction, and $u_{r}^{ƒ}$ is the unit vector parallel to the separation distance. The exponents *p* and *q* were assigned values, respectively, of 6 and 3 that control the strength of cell-cell interactions.

##### Extracellular environment

The extracellular matrix (ECM) environment is assumed to be composed only of collagen posts, which are modeled as fixed elastic blocks that are randomly distributed around the cell cluster. Varying the density of collagen blocks allows one to vary the collagen density within the ECM. In this model, only physical interaction forces between cell and ECM are captured, and therefore does not consider active interactions such as the potential lysis of collagen by migrating cells. As such, cells have minimal interactive forces with the collagen blocks, consisting of small repulsion forces at the interface of cell surface-collagen surface, to ensure no spatial overlap. These interactions are modeled with a Lennard-Jones potential as described in Equation 6, with exponents *p* and *q* with values of 2 and 0 (no cell-ECM adhesion), respectively.

##### Model configuration

The initial configuration of the model is constituted by 152 cells arranged in a circular collective within an ECM environment represented by collagen blocks. Cell motility is represented by the propulsion vector *v*_0_, which is homogeneous in magnitude across the cell collective, and with *v*_0_ increasing as the cells comprising the collective become more and more motile. All the parameters described above are homogeneous across the cell population with values specified in the next section, the only major differentiator being the random motility orientation for each cell. Specifically, the initial model configuration is generated as follows. A number of points (n = 340) are seeded in a rectangular environment (261 × 160 pixels). Following a Poisson disk sampling to ensure a more uniform distribution of the generated points, a Voronoi tessellation is created based on these cell centers. The generated polygons are then shrunk isotropically to ensure a distance *ε*> 0 between all neighboring edges. The cell membrane is simulated by placing 25 equidistant points (indicated as *i*, *j*, *k* in the sections above) on each polygon’s sides. A disk of radius 73 pixels is used to sample the cells within a circular collective, leaving the remainder of the environment empty. This reduces the number of cells from the initial 340 in the whole environment to a total of 152 cells within the circular disk. The simulated ECM environment is obtained by seeding a specific number of points (depending on the desired collagen density) in an extended environment (520 × 320 pixels) and generating spherical collagen blocks, with a fixed radius (7 pixels) and a fixed number of exterior points (12 points). Starting from this initial configuration, the model simulates the invasive behavior of the cell collective over time for a total of 120 time steps per simulation.

##### Simulation set-up

The simulation is advanced by adopting a semi-implicit Euler scheme:

(Equation 7) $$\begin{array}{l} v_{t+\Delta t}=v_{t}+a_{t}\Delta t,\\ x_{t+\Delta t}=x_{t}+v_{t+\Delta t}\Delta t, \end{array}$$

At time *t*+*Δt*, the velocity of any point on the cell membrane *v*_*t*+*Δt*_ is computed using the previous timepoint’s velocity *v*_*t*_ and acceleration *a*_*t*_. Acceleration for each cell is the total force applied on the cell at time *t* divided by the mass. For simplicity, the mass was taken to be 1 for all cells. Following the computation of the updated velocity, the next position (*x*_*t*+*Δt*_) of each point on the cell membrane is computed to advance the simulation. The whole system is damped by a parameter *v*_*decay*_ that serves as a correction for the acceleration generated by errors in the advancing method. This damping correction is implemented by updating *v*_*t*+*Δt*_ (S8-1) with 1−*v*_*decay*_ at each time step. Based on preliminary simulations, here we used *k*_*m*_=0.25, *l*_*ij*,0_=1.48, *γ*_*m*_=5, *k*_*b*_=2×10^−3^, *k*_*a*_=1×10^−3^, *a*_0_=10^2^, *k*_*f*_=5×10^−4^, *γ*_*f*_=0.58, *k*_*LJ*_=0.75, *r*_0_=1, *v*_*decay*_=10^−4^, all expressed in arbitrary units (A.U.).

##### Phase diagram simulation and robustness testing

To investigate the effect of varying cell motility and collagen density on spheroid invasive behaviors, we varied the cell propulsion *v*_0_ in the range [0.6, 1.18], with a step increment of 0.02 to simulate the effect of increased cell motility. Meanwhile, the effect from the surrounding ECM that the cell collective experiences is tuned by modifying the spatial density of collagen blocks. For each combination of cell motility and collagen density, the 95^th^ percentile of radial cell positions (**p**^95th^) at the end of each simulation was computed and the cumulative distribution of **p**^95th^ resulting from all simulations (Figure 5C) was used as a metric to distinguish between material phases (solid, liquid or gaseous) exhibited by the invading cell front. By using **p**^95th^, we chose to examine only the position of the 5% outermost cells as the minimal fraction of the cell collective that is needed to capture the periphery of the tumor near the cell-ECM interface. In addition, **p**^95th^ provides the most straightforward way to identify what type of phase transitions occurred in the cell collective. Based on the resulting distribution, the thresholds for each of the phases were set as the 34^th^ percentile for the liquid phase, and as the 64^th^ percentile for the gaseous phase (Figure 5C). All simulations were run in 10 replicates, each using different sets of collagen post distribution and cell motility orientation. Each data point from the phase diagram in Figure 5D represent the averaged result from these 10 configurations.

To assess the effect of the arbitrary thresholds chosen based on the radial distance from the center of the cell collective on the identification of distinct material phases, we performed two separate tests. First, we performed a sensitivity analysis aimed at assessing the influence of different threshold values on the phase diagram. We found that the phase diagram is stable, and not sensitive to a number of threshold changes (Table S2). Second, we assessed whether a criterion based solely on radial distance reflects differences in cell shape, thereby separating different material phases. Based on a fixed initial configuration of both cells and collagen, we ran a new set of simulations in correspondence of all combinations of cell motility and collagen density. For such combinations, which reflect all the points on the phase diagram, we assessed the material phase based on the radial distance criterion and calculated the average cell shape index. This average cell shape, defined as SI=*perimeter*/$\sqrt{\mathrm{area}}$ for cells in 2D, is calculated for cells within a 95% radial distance from the center of each collective, and allowed characterization of the bulk of the collective that is not fully captured using the radial distance criterion. By comparing the mean SI of cell collectives that are categorized as solid-like or liquid-like by the radial distance criterion, we found that liquid-like cell collectives are on average more elongated and have more variables shapes (SI^liquid^ = 4.75 ± 0.35) with respect to their solid-like counterparts (SI^solid^ = 4.50 ± 0.11, p = 1.95 × 10^−6^, H0: SI^liquid^ = SI^solid^), as expected from modeling studies (Bi et al., 2016). It should be noted that a mean SI is not computed for individual gas-like cells, as it is defined only for multicellular collectives in which individual cells are fully surrounded by neighboring cells.

### Quantification and statistical analysis

#### Measurement of micro-spheroid dynamics

MCF-10A cells were stably transfected with GFP-linked nuclear localization (NLS-GFP). To track the movement of individual cells, micro-spheroids at early (3–5 days) and late (7–10 days) stage of growth were imaged every 10 min for 12 h in a customized incubator (37°C, 5% CO_2_, and 95% humidity) on a confocal microscope (Leica, TCL SP8), with acquisition of fluorescent and bright-field image z-stacks (Figures 1A and 1B). Analysis of cell migratory dynamics was done based on the obtained image z-stacks according to the procedures outlined below, implemented in custom Matlab programs (cf. *nuclei identification*). Using an adaptation of previously published methods (Crocker and Grier, 1996), the 3D coordinates of each cell nucleus were identified at each frame based on maximum intensities from the fluorescent images. Cell trajectories were constructed through minimization of overall nuclear displacements between sequential frames. Cell positions from each time point were aligned to the micro-spheroid geometric center at the initial time point *t*_0_ to account for any translational motion that may have occurred due to stage drift. To separate the contribution of Coherent Angular Motion (CAM) of the early micro-spheroid from migratory dynamics due to individual cell rearrangement, we solved for the rigid rotational transformation *ω* that best reproduced the changes occurred in cell nuclei positions (*p*(*x*,*t*)) between z-stacks during the time interval *Δt*. *ω*is defined as the rotation that minimized the metric d(ω|t)=p(x,t+Δt)−R(ωΔt)p(x,t). This rigid rotational motion from CAM is removed from cell trajectories before further calculations of cellular dynamics. Root mean squared (RMS) speeds for each cell were calculated using the instantaneous velocity vectors. Mean squared displacements (MSD) were computed as a function of time interval, $MSD\left(\Delta t\right)=\left\langle {\left|p\left(x,t+\Delta \text{t}\right)-p\left(x,t\right)\right|}^{2}\right\rangle$. Here, ⟨…⟩denotes an average over time, with overlap in time intervals. Similarly, the overlap parameter *Q*_*s*_ was defined as the time average $Q_{s}\left(\Delta t,r\right)=\left\langle q_{s}\left(\Delta t,t,r\right)\right\rangle$, where $q\left(\Delta t,t,r\right)=1-\beta {\left\|p\left(x,t+\Delta t\right)-R\left(\omega \Delta t\right)p\left(x,t\right)\right\|}^{2}$. Following the approach of Palamidessi et al. (2019), we calculated the relaxation rate of the self-overlap parameter *Q*_*s*_(*Δt*,*r*) by means of $\Gamma \left(r\right)=\left[Q_{s}\left(\;\Delta t_{0},r\right)-Q_{s}\left(\;0,r\right)\right]/\Delta t_{0}$, which measured the local tissue fluidity at each radial position *r*.

#### Measurement of macro-spheroid dynamics

A customized spinning disk confocal setup equipped with an environmental chamber (37°C, 5% CO_2_, 80% relative humidity) was used for imaging spheroid invasion in collagen. For each spheroid within a 6-well plate, differential interference contrast (DIC) images were collected every 10 min for 48 h as 3 × 3 tiled, 200 μm z-stacks. A 10× air objective was used to image large areas (1450.3×1450.3 μm) with a resolution of 1.126 μm/pixel. Automated stitching based on global optimization of the 3D stacks (Preibisch et al., 2009) was carried out using ImageJ (v. 1.52g, National Institute of Health, Bethesda, MD). Minimum intensity projections of the stitched DIC time-lapse data were used to visualize the 3D datasets as 2D Videos while maximizing the image contrast (dark spheroid/cells on a light background). These DIC movies were imported in Matlab, where images from individual time frames were down-sampled by 50% (2.252 μm/pixel) to optimize the processing speed. De-jittering of movies was achieved using an optimized image registration algorithm (Guizar-Sicairos et al., 2008). Temporal evolution of the projected macro-spheroid area was segmented using Otsu’s method, where the optimal threshold was calculated over three consecutive frames to smooth changes in area between frames. The resulting binary mask allowed us to separate between the spheroid interior (i.e., main spheroid) and exterior (i.e., single cells migrating in collagen, if any). To estimate the direction and speed of collective migration within the macro-spheroid, we employed Farneback’s optical flow method using the Matlab function *estimateFlow* with the option *opticalFlowFarneback* and a Gaussian filter size of 8 pixels while keeping all the other parameters to their default values. At each frame, velocity vectors are obtained for seed points spaced 2 μm apart that are inside the spheroid mask. To account for the effect of collective motion from spheroid growth on migratory dynamics, we estimated spheroid radial growth rate through tracking of change in spheroid mask area over time. This velocity was interpolated for each seed depending on its location within the spheroid, and were removed from the velocity vectors before RMS speed calculations. Single cells lying outside the spheroid mask were detected based on local intensity minima at each frame, and tracks constructed by minimizing displacements of cells between consecutive frames. Both collective and single cell velocity vectors were smoothed with a moving average filter of 3 frames with unity weighting in the temporal domain. RMS speed were calculated for each cell/seed using the instantaneous velocity vectors.

#### Cell shape characterization

We used a customized analysis procedure in Matlab to characterize the cell shapes from both micro- and macro-spheroids. Steps in the analysis pipeline are illustrated in Figure S2 and described below.

##### Nuclei identification

Nuclei centers were identified based on local intensity maxima from fluorescent image stacks (DAPI or GLP-NLS) with adaptations of previously published methods (Crocker and Grier, 1996; Toyoshima et al., 2016). In brief, images were preprocessed with Matlab function *medfilt2* and *smoothn* to minimize background noise and smooth out intensity variation within each nucleus. To reduce noise, background intensity outside the spheroid were removed by thresholding for the lowest 10% of signal intensities. Local intensity maxima were then identified from each 2D image slice, and a connected component analysis and a search radius of 5 μm in 3D was used to identify nuclei centers. Accuracy of nuclei identification of each spheroid was assessed via visual inspection of the overlay between identified nuclei positions with fluorescent nuclei image stacks (Figure S2B). Further validation of this nuclei identification algorithm was carried out by comparing algorithm-identified cell counts with the manual counts obtained using a hemocytometer after spheroid dissociation via trypsinization. Validation was performed for macro-spheroids seeded at different sizes for both MCF-10A and MDA-MB-231 cells (Figure S2G).

##### Spheroid boundary segmentation

Spheroid boundaries were identified from either bright-field microscopy (for micro-spheroids) or multiphoton SHG (for macro-spheroids) z-stacks. The spheroid boundary was identified in a two-step (coarse and fine) segmentation process, adapting previously published protocols (Bradbury and Wan, 2010; Tse et al., 2009). Image z-stacks were preprocessed with intermediate steps including *histogram equalization*, *wiener* (8 × 8 pixels) and *median* (5 × 5 pixels) filters to enhance image contrast. Initial coarse segmentation was performed using the watershed algorithm on the gradient image of the resulting image z-stacks, supplemented with nuclei position information. The fine segmentation step involved adaptive k-means clustering based on variation in pixel intensities in the output image from the coarse segmentation step. The number of clusters segmented was determined automatically based on the distance between new and existing cluster centers from the previous iteration. The final spheroid boundary was taken as the data cloud outline of the spheroid cluster that formed a connected region with the largest volume. Validation was done by visual inspection of the overlay between identified spheroid boundaries with the raw image z-stacks (Figure S2C).

##### Bounded Voronoi tessellation

Voronoi tessellation of the nuclei centers were based on the observation by Voigt and Weis(2010), who have shown that the vertices of each Voronoi cell are solution to sets of linear inequalities indexed by their nuclei centers. Delaunay triangulation of the nuclei centers was used to identify the Voronoi neighbors for each cell. The spheroid boundary data cloud was then downsampled to 5% while maintaining the shape of the spheroid. The set of linear inequality for each cell was then constructed from the union between the perpendicular bisectors between edges connecting the cell and its Voronoi neighbor; and the spheroid boundary identified from segmentation. Some cell-free regions in the macro-spheroid core caused the Voronoi tessellation to give falsely large cell volumes that included these regions, a double thresholding was applied to discard these cells. Voronoi cells were discarded if cell volumes exceeded 3000 μm^3^ or maximum distance of a cell to its immediate neighbors was in the top 5% of average maximum distance between all neighbors (Figure S2F).

#### Statistical analysis

All of the data was analyzed in Matlab (R2019a, Mathworks, Natick, MA). Experimental data are presented as mean ± STD unless otherwise stated. One-tailed t-tests were used to assess differences between spatial regions (core vs. periphery) and stages of evolution (early vs. late) of the micro-spheroid. A one-way ANOVA was used to test structural and mechanical differences due to collagen concentration and radial differences in cell shape and RMS speed in the macro-spheroid. Post-hoc pair-wise comparisons were performed using the Bonferroni correction. Unless otherwise stated, p<0.05 was considered statistically significant. All of the statistical details of experiments can be found in the figure legends.

##### Maximum likelihood estimation (MLE)

We fitted SI distributions to the *k-gamma distribution* using maximum likelihood estimation as described previously (Atia et al., 2018). In brief, for a dataset $\left\{x_{i}\right\}i=1,\ldots ,\text{N},$ the likehood function to fit is $L\left(k\right)=\prod_{i=1}^{N}PDF\left(x_{i};k\right)$, where $\text{PDF}\left(x;k\right)=k^{k}x^{k-1}e^{-kx}/\text{Γ}\left(k\right)$ is the *k-gamma* probability density function. For our purposes, the SIs were shifted and normalized, and defined as $x=\left(SI-SI_{min}\right)/\left(\bar{SI}-SI_{min}\right),\;$ with *SI*_*min*_=5.413 based on Merkel and Manning (2018).

## Data Availability

• All data reported in this paper will be shared by the lead contact upon request. • All original code has been deposited at Zenodo and is publicly available as of the date of publication. DOIs are listed in the key resources table. • Any additional information required to reanalyze the data reported in this paper is available from the lead contact upon request.

## Supporting citation

The following references appear in the Supplemental Information: Mirtich, 1996.
