# Supplementary material for: A novel jamming phase diagram links tumor invasion to non-equilibrium phase separation
Source: iScience. 2021 Oct 12;24(11):103252. doi: 10.1016/j.isci.2021.103252 (PMC8564056; doi:10.1016/j.isci.2021.103252)
Supplement: Document S1. Figures S1–S9 and Tables S1 and S2 [file mmc1.pdf]

iScience, Volume *24*

Supplemental information

A novel jamming phase diagram links tumor invasion to non-equilibrium phase separation

Wenying Kang, Jacopo Ferruzzi, Catalina-Paula Spatarelu, Yu Long Han, Yasha Sharma, Stephan A. Koehler, Jennifer A. Mitchel, Adil Khan, James P. Butler, Darren Roblyer, Muhammad H. Zaman, Jin-Ah Park, Ming Guo, Zi Chen, Adrian F. Pegoraro, and Jeffrey J. Fredberg

SUPPLEMENTARY FIGURES

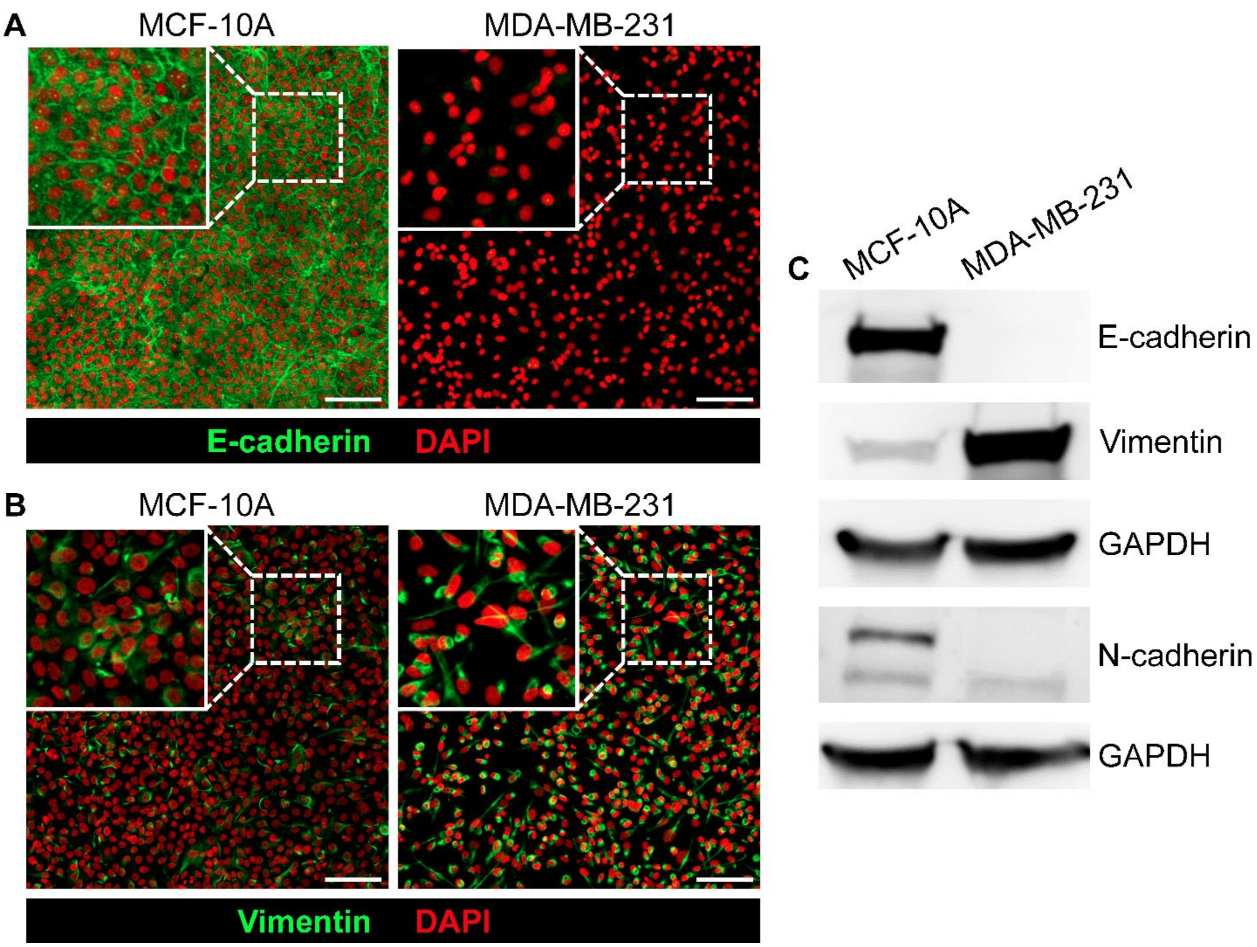

Figure S1. Characterization of MCF-10A and MDA-MB-231 cell lines. Related to STAR Methods. Representative immunofluorescence (IF) staining for (**A**) E-cadherin and (**B**) Vimentin shows that MCF-10A cells display predominantly epithelial characteristics while MDA-MB-231 cells show predominantly mesenchymal characteristics. Scale bars represent 100 µm. (**C**)Western blot analysis of MCF-10A and MDA-MB-231 monolayers confirms IF findings. E-cadherin is expressed only in MCF-10A but not MDA-MB-231 cells, while vimentin is expressed at a low but detectable level in MCF-10A cells and at a high level in MDA-MB-231 cells. Both cell types show expression of N-cadherin, with higher expression in MCF-10A cells. Together these data indicate that MDA-MB-231 cells are fully mesenchymal, while MCF-10A cells are mostly epithelial but with some aspects of a partial EMT phenotype, as is typical for this model epithelial cell line.

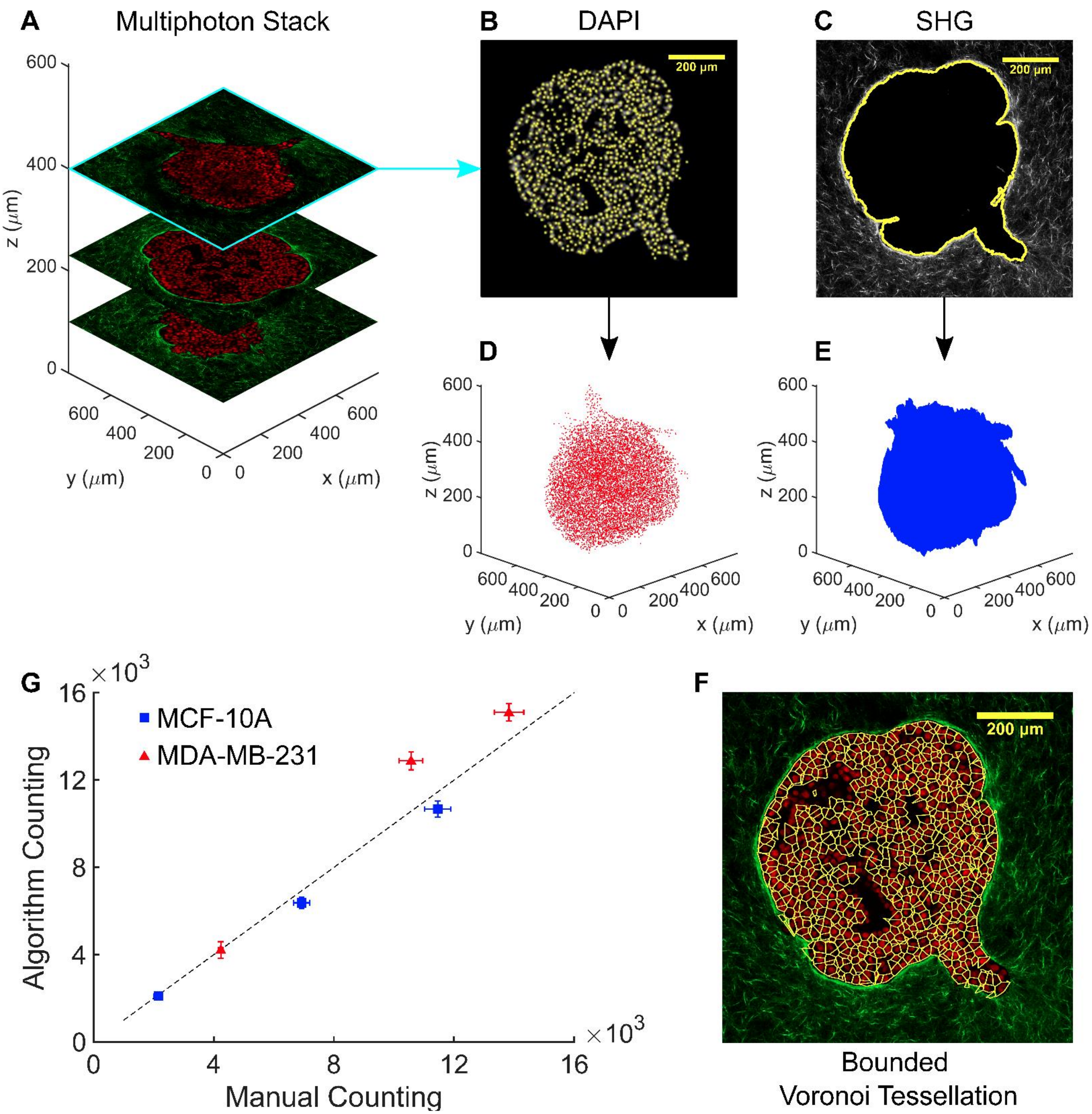

Figure S2. Workflow for 3D cell identification and cell shape estimation in multicellular spheroids. Related to Figures 1-3. Here, the workflow is illustrated for a representative MCF-10A macro-spheroid in collagen, but was also applied to MCF-10A micro-spheroids and MDA-MB-231 macro-spheroids alike. (**A**) A representative multiphoton stack of a MCF-10A macro-spheroid cultured in 2 mg/ml collagen is split into two channels, carrying separate signals: (**B**) fluorescence from DAPI-stained nuclei and (**C**) second harmonic generation (SHG) from collagen. In the case of MCF-10A micro-spheroids in Matrigel and Alginate, the two channels are given by fluorescent and bright-field images (cf. Figure 1). The two channels are processed, respectively, to identify (**D**) the 3D position of cell nuclei and (**E**) the 3D spheroid boundary using a custom

algorithm. Note that the representative slices (**B-C**) displaying 2D projections of identified nuclei centers and spheroid boundary are shown overlapped onto corresponding multiphoton images (all nuclei centers intersecting the image plane are shown). (**F**) Data on nuclei location and spheroid boundary are combined to generate a bounded Voronoi tessellation that partitions the spheroid into individual cells, from which cell volume and shape metrics are derived. Here, the 2D cross-section view of resultant Voronoi cells are shown superimposed on the corresponding multi-photon image. We note that empty spaces within the spheroid (cf. Figure S6) as well as its neighboring cells are excluded from further analysis to minimize local overestimation of cell shape (**G**) Our custom 3D nuclei detection algorithm was validated by comparing the number of cell nuclei counted by the algorithm in fixed and cleared spheroids with the number of cell nuclei estimated for spheroids that were dissociated via trypsination and manually counted using a hemacytometer. Over a wide range of spheroid sizes, using both the MCF-10A (blue squares) and MDA-MB-231 (red triangles) cell lines, manual and algorithm counts lie close to the identity line (dashed black line). Spheroids used for counting validation were formed starting from a variety of cell numbers (from ~10^3 to ~10^4) at the time of seeding which increased (to ~2×10^3 to ~1.5×10^4) over the course of the 48 hours during which spheroid formation occurred (cf. Figure S6). Both cell couting methods used spheroids from the same batch and cells at the same passage. Data are presented as mean ± SEM (n = 6-10 for manually counted spheroids and n = 3-4 for automaticaly counted spheroids of all sizes).

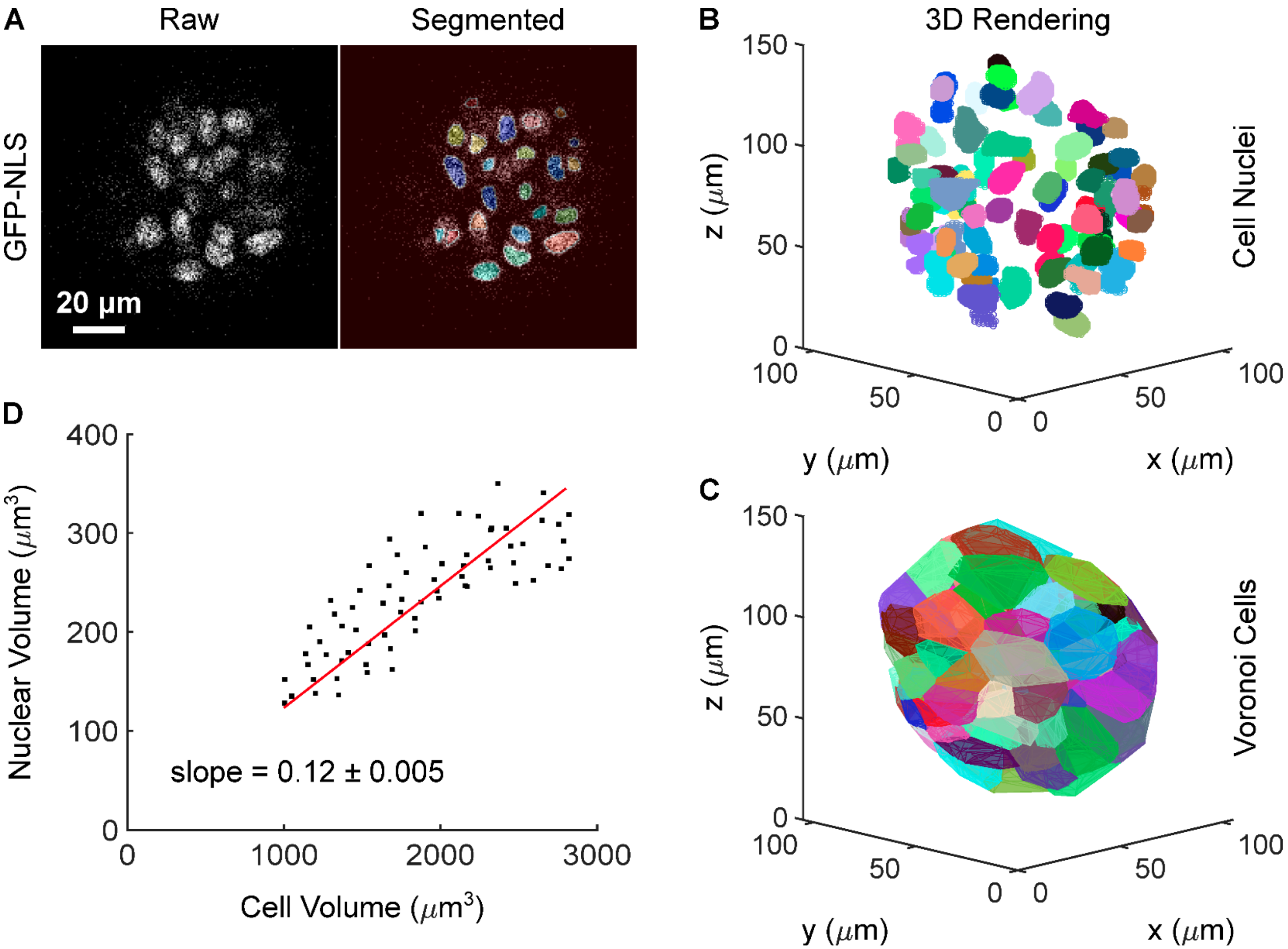

Figure S3. Cell volume from 3D Voronoi tessellation tracks nuclear volume in multicellular spheroids. Related to Figure 1. (**A**) Equatorial cross-section of a representative confocal microscopy image from an early GFP-NLS labelled MCF-10A micro-spheroid juxtaposed to the segmented image, in which each identified nucleus is labeled with a different color. (**B**) 3D rendering of an early MCF-10A spheroid in which cell nuclei are reconstructed from segmentation of confocal stacks. (**C**) 3D rendering of the same spheroid in which Voronoi cells are obtained using the workflow outlined in Figure S2. (**D**) Voronoi cell volume plotted against nuclear volume for individual cells show that the two measures are linearly related (R^2=0.95).

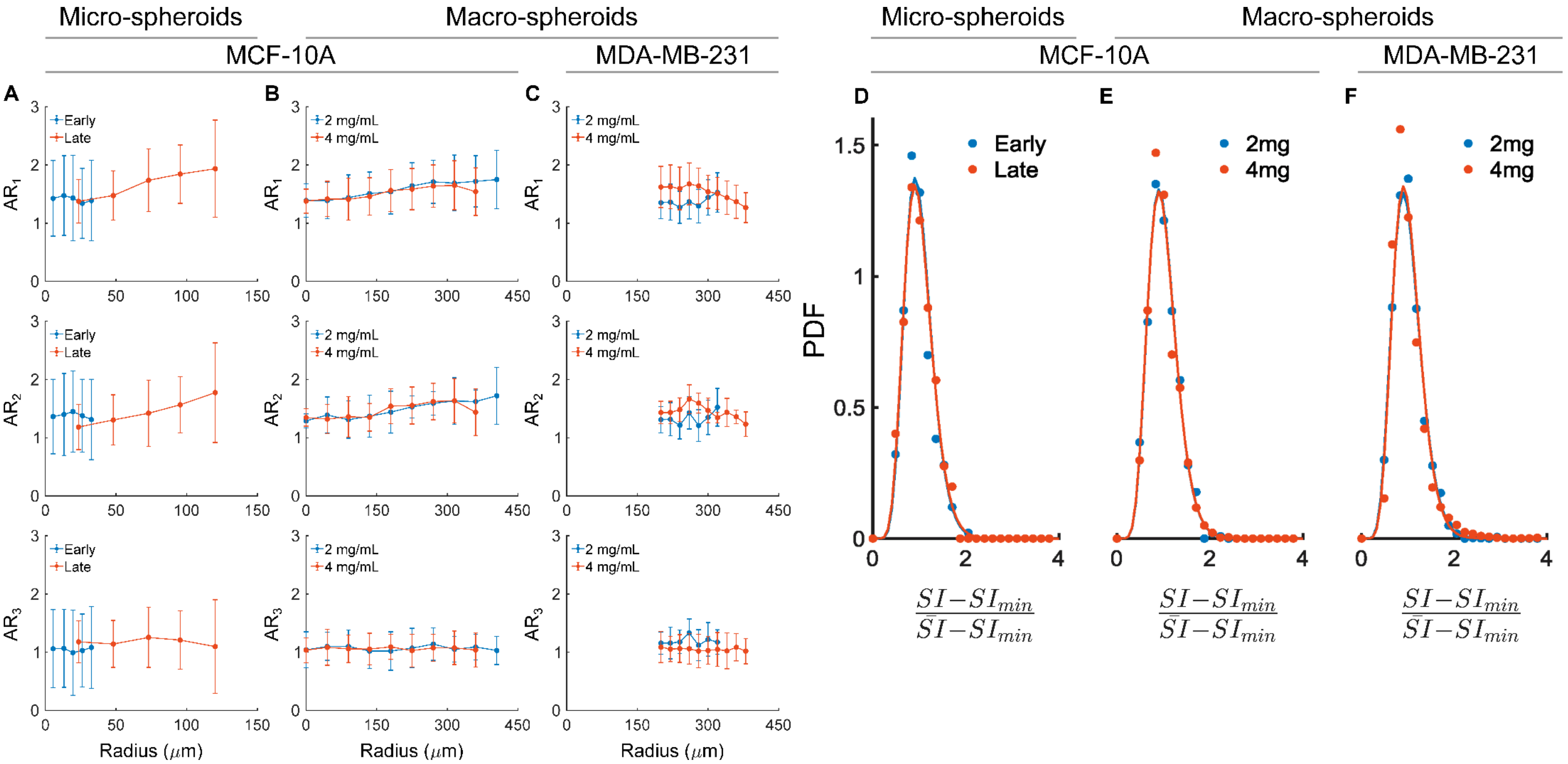

Figure S4. Aspect Ratio (AR) and Shape Index (SI) distributions within multicellular spheroids. Related to Figure 1. Following (Atia et al., 2018), we used tesselletad Voronoi cells to quantify cell shape in terms of AR, which is closely associated with the second moments of inertia **I** and can be described using the diagonal terms of the second-order tensor $\mathbf{I} = diag\{I_1, I_2, I_3\}$. We treated each cell as a polyhedron of uniform density, and calculated respective moment of inertia tensors according to (Mirtich, 1996). The three AR metrics, arranged in descending order, are $AR_1 = I_1/I_2$, $AR_2 = I_1/I_3$ and $AR_3 = I_2/I_3$. In addition to serving as a structural signature of unjamming, respective change in the three ARs also inform about nature and orientation of cell shape changes. (**A**) Upon MCF-10A micro-spheroid growth from early to late stage, both AR_1 and AR_2 show development of increasing radial gradients towards larger AR for cells in the periphery of late stage spheroids. The increase in AR are consistent with increase in cell

shape index (cf. Figure 1), and support the existence of more elongated cells – that is, unjammed – peripherial cells. In addition, the radial increase in both AR_1 and AR_2 highlights the existence of more rounded cells in the spheroid center (AR near 1) and more rod-like cells at the spheroid periphery, in line with interpretation of cell polarization outward to invade into the matrix. (**B**) Similar, but less pronounced, gradients can be seen in MCF-10A macro-spheroids cultured in collagen, where AR_1 and AR_2 increase towards the spheroid periphery only for speroids embedded in 2 mg/ml collagen, that is, for spheroids that undergo local unjamming with invasion. (**C**) Instead, a small radial decrease in AR_1 is seen for cells from MDA-MB-231 macro-speroids in 4 mg/ml collagen, which suggests a transtion to more rounded cell shapes in correspondece of the invasive protrusions, thus supporting the notion of a confinement-induced jamming transition. For each Voronoi cell, we computed the SI as $surface/volume^{2/3}$ and fitted SI distributions to the *k-gamma distribution* using maximum likelihood estimation (Atia et al., 2018). Each distribution is generated by pooling data from all experiments and dividing it into 50 bins. Here we present the experimentally obtained PDFs as data points (circles) and show their respective best-fitting k-gamma distributions (solid lines of the corresponding color). (**D**) MCF-10A micro-spheroids shape distributions at the early and late stage fit for a k-value of 10.06 ± 0.05 and 10.01 ± 0.07, respectively (p=0.32). (**E**) MCF-10A macro-spheroids shape distributions in 2 and and 4 mg/ml collagen fit for a k-value of 10.14 ± 0.17 and 10.20 ± 0.11, respectively (p=0.51). **(F)** MDA-MB-231 macro-spehoids shape distributions in 2 and 4 mg/ml collagen fit for a k-value of 10.38 ± 0.10 and 10.26 ± 0.15, respectively (p=0.24). Across cell types, experimental preparations, and invasion phenotypes, the distribution of cell shapes robustly followed a k-gamma distribution. Surprisingly, there was no statistically significant difference between the k-value that describe these distributions (H0: no difference in k=values between respective conditions). Average k-value across cell types and spheroid preparations is 10.19 ± 0.1, consistent with the k-value range from the theory of 3D inert matter packings from (Aste & Di Matteo, 2008), and suggests that variations in individual cell shapes are perhaps due to short ranged interaction between immediate neighbors.

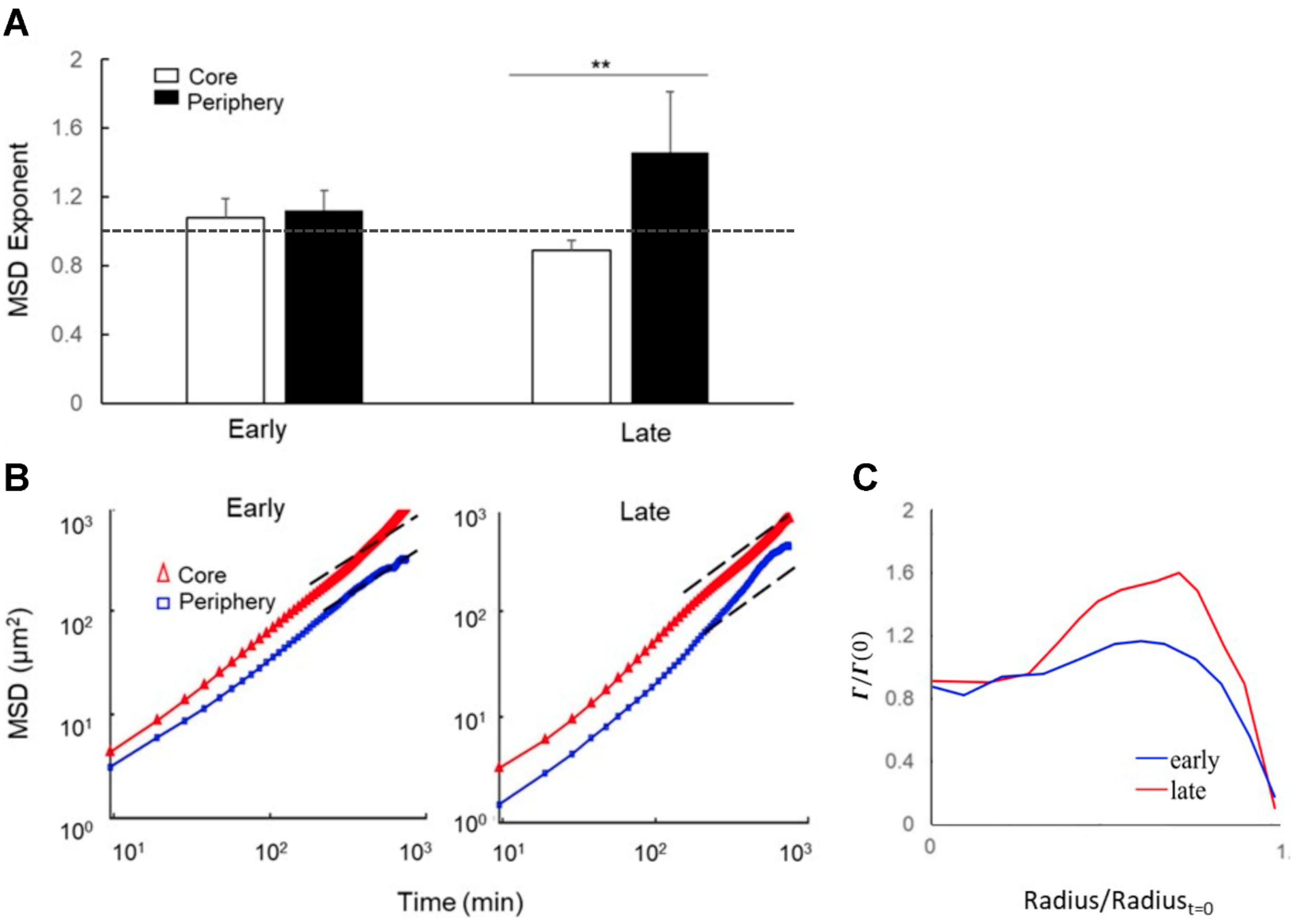

Figure S5. Spatio-temporal dependence of cellular motion during micro-spheroid growth and invasion. Related to Figure 1. (**A**) The exponent of mean squared displacements (MSD) shows that cellular motions are diffusive and homogenous in the early stage micro-spheroid ($p=0.67$), while spatial dependence in cell motion is clear in the late stage micro-spheroid ($p<0.01$). In the late stage micro-spheroid, celllular motion is sub-diffusive ($p=0.051$, H0: exponent not different from 1) in the spheroid core suggestive of caging, while periphery cells are clearly super-diffusive. MSD exponent = 1 indicating diffusive behavior is shown with a dashed line to guide the eye. MSD exponents are obtained from linear fitting of MSDs at later times (500 to 1000 minutes) as a function of time on a log-log plot. Core regions are defined as radius < 50% of initial micro-spheroid radius, and those above as periphery region. Data are presented as mean ± standard deviations (n=5 for micro-spheroids at early and late stage respectively). (**B**) Time and ensemble averaged MSD of cell trajectories from all spheroids over the observation window. The black dashed lines show a slope of 1 (MSD exponent = 1, suggestive of diffusive motion) to guide the eye. (**C**) Average relaxation rates of the self-overlap parameter (STAR Methods) show a stronger radial dependence for late stage micro-spheroids with respect to early stage micro-spheroids. This larger relaxation rate at the spheroid periphery indicates increased celluar rearrangement and tissue fluidization.

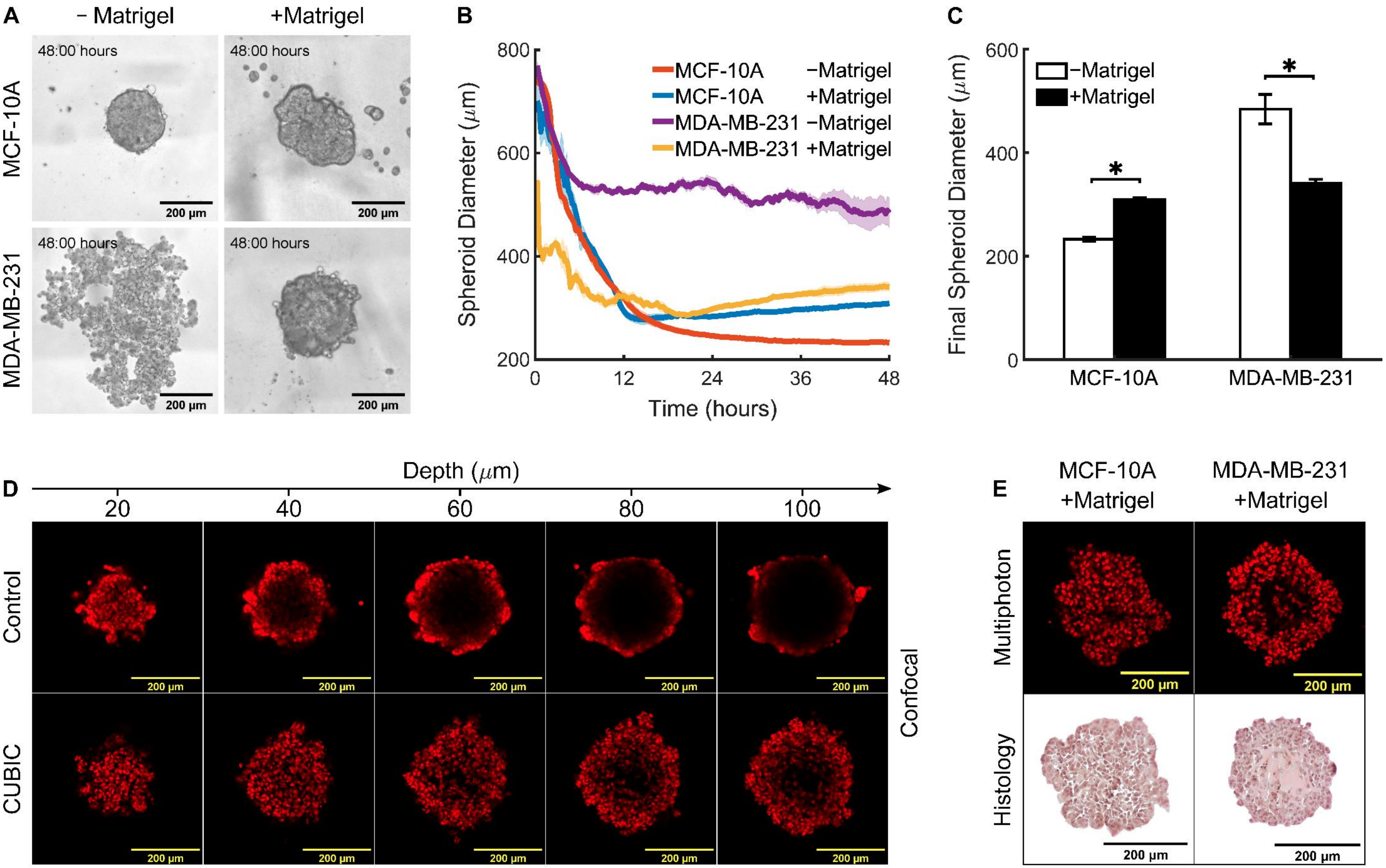

A
− Matrigel
+Matrigel
MCF-10A
MDA-MB-231
48:00 hours
200 μm
B
MCF-10A −Matrigel
MCF-10A +Matrigel
MDA-MB-231 −Matrigel
MDA-MB-231 +Matrigel
Spheroid Diameter (μm)
Time (hours)
C
−Matrigel
+Matrigel
Final Spheroid Diameter (μm)
MCF-10A
MDA-MB-231
D
Depth (μm)
20
40
60
80
100
Control
CUBIC
Confocal
E
MCF-10A +Matrigel
MDA-MB-231 +Matrigel
Multiphoton
Histology
200 μm

Figure S6. Formation and 3D imaging of MCF-10A and MDA-MB-231 macro-spheroids. Related to Figures 2-4. (**A**) Representative DIC images of macro-spheroids formed in low attachment conditions for 48 hours starting from approximately 10^3 cells in presence (+) or absence (-) of 2.5% Matrigel diluted in cell culture media. The presence of Matrigel leads to MCF-10A macro-spheroids that are larger and less regular in shape with respect to Matrigel-free controls. On the other hand, MDA-MB-231 cells form only loose aggregates in absence of Matrigel while compacting effectively in its presence. (**B**) Time-course of spheroid compaction over the course of 48 hours. The equivalent spheroid diameter *d* is calculated from the total cell area *A* thresholded from DIC time-lapse movies as $d = \sqrt{4/\pi \cdot A}$. (**C)** Spheroid diameter at 48 hours of compaction quantifies the differences caused by the presence of Matrigel in the different cell lines. Both cell lines form spheroids of similar sizes in the presence of Matrigel (black bars). Data are presented as mean ± SEM (n = 3 for all groups). * indicates statistical significance at $p < 0.05$. (**D**) 3D scanning confocal microcopy images of representative MDA-MB-231 spheroids after fixation (Control) or fixation followed by optical clearing (CUBIC), shows that DAPI-stained nuclei (red) in the spheroid core are clearly visible only after optical clearing. The depth of the confocal stacks represents the distance from the spheroid surface. (**E**) Representative equatorial cross-sections of MCF-10A and MDA-MB-231 spheroids generated in presence of Matrigel show the distribution of cell nuclei using two methods: multiphoton imaging of DAPI-stained cell nuclei after CUBIC clearing (top), and van Gieson's stained histology slides (bottom). Note that the hollow core of MDA-MB-231 spheroids is not filled by cells (identified by the dark nuclei) but rather is occupied by matricellular proteins (shown in red).

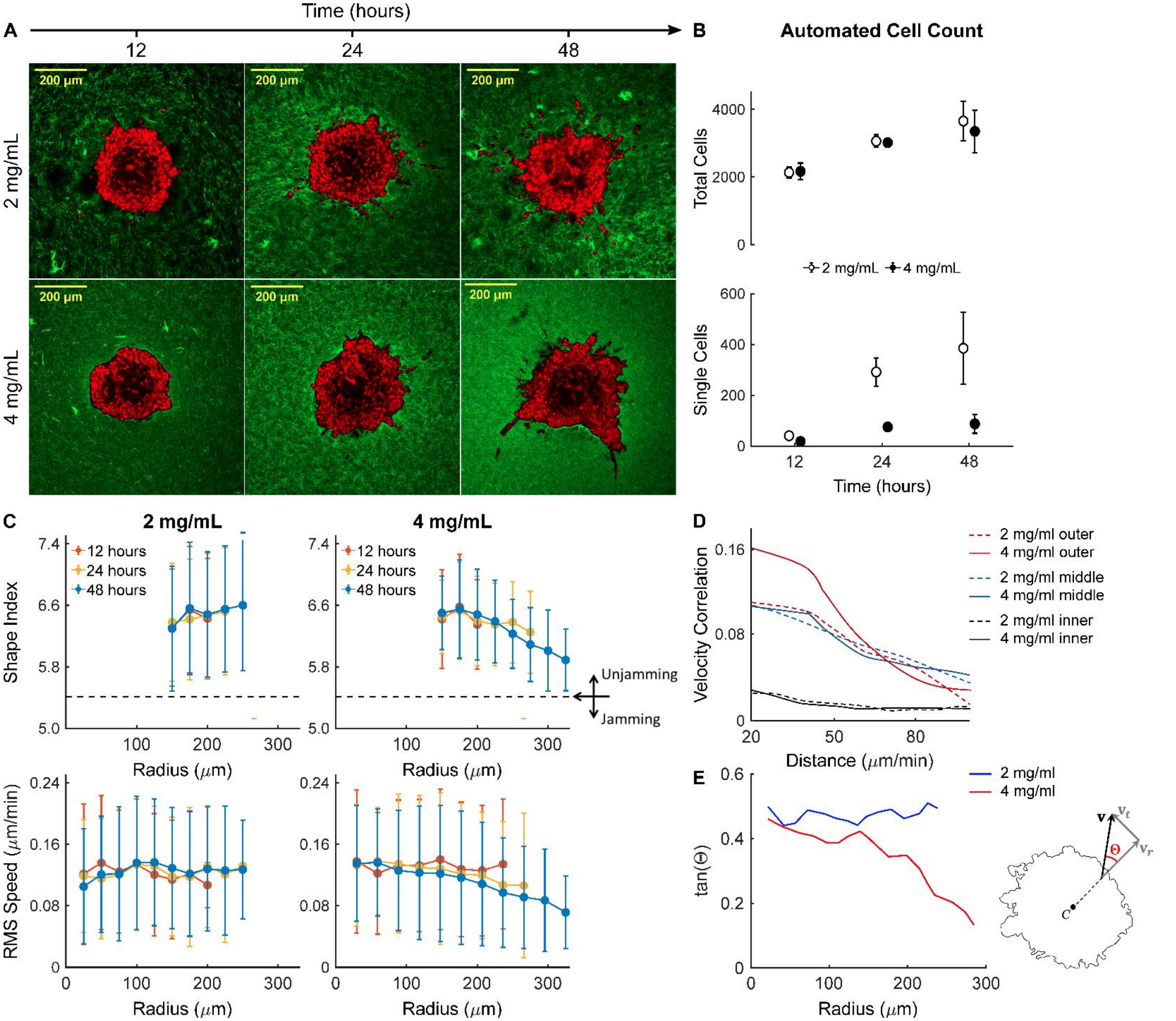

Figure S7. **Over time, MDA-MB-231 macro-spheroids in high density collagen experience confinement-induced jamming at the invasive front. Related to Figure 3.** We carried out a time-lapse experiment, including continuous DIC imaging as well as fixation and optical clearing for multiphoton microscopy, for MDA-MB-231 spheroids at distinct time points. (**A**) Representative equatorial cross-sections of multiphoton images display DAPI-stained cell nuclei (red) and collagen fibers from SHG (green) from MDA-MB-231 spheroids embedded in 2 and 4mg/ml collagen and imaged after 12, 24, and 48 hours. (**B**) Total and single cell counts over time show that cell proliferation is quite similar between spheroids embedded in various collagen densities, while there is a significantly higher single cell escape over time in spheroids embedded in 2 mg/ml collagen. (**C**) Temporal evolution of cell shape (top) and migratory speed (bottom) in spheroids embedded in 2 mg/ml (left), and 4 mg/ml (right) collagen. In 2 mg/ml collagen, MDA-MB-231 cells that remain within the primary spheroid maintained homogenous radial distributions for both cell shape and RMS speed. In contrast, in 4 mg/ml collagen, MDA-MB-

231 cells within the primary spheroid have similar homogenous distributions until 12 hours of culture (SI: p=0.31, RMS speed: p=0.58, H_0: 2 mg/ml is not different from 4 mg/ml), but progressively develop radially decreasing trends for both cell shape and RMS speed at 24 and 48 hours (SI: 95%CI = [-0. 36, -0.78], p=0.05, RMS speed: 95%CI = [-0.038, -0.069], p=0.003, H_0: no differences exist between the spheroid core and periphery at 48 hours). Peripheral cells from spheroids invading in 4 mg/ml collagen became signficantly smaller in cell shape and slower in RMS speed (SI: 95% CI=[-0.38,-0.80], p=0.04; RMS speed: 95%CI=[-0.032,-0.075], p=0.02, H_0: no differences exist in peripphery cells between 2 mg/ml and 4 mg/ml collagen). The critical cell shape (SI = 5.4) predicted by a static Voronoi model of cellular jamming (Merkel & Manning, 2018) is indicated by a horizontal dashed line, with increasing values indicative of unjamming and decreasing values indicative of jamming. Overall, we observe that ECM confinemnt induces a jamming transition, or confinement-induced jamming, at the invasive front. Statistical tests between groups and conditions were performed using a one-way ANOVA, with core and periphery cells determined based on distance from the spheroid center (core: r <200; periphery: r > 200) (**D**) Spatial dependence in velocity correlations calculated from optical flow trajectories for spheroids embedded in 2 mg/ml (dashed lines) and 4 mg/ml (solid lines) collagen.Correlations in migratory motion are spatially divided between inner (black lines, r < 100 um), middle (blue lines, 100< r< 200 um) and outer (red lines, r > 200 um) regions. Velocity correlations remain low regardless of collagen concentration in the inner region which suggests low coordination in migratory motion. Only at the invasive periphery of spheroids in 4 mg/ml collagen, we observe elevated velocity correlations suggesting that neighboring cells display elevated coordination of motility under high ECM confinement with respect to cells in low ECM confinement. (**E**) Mean ratio between tangential ($\boldsymbol{v_t}$) and radial ($\boldsymbol{v_r}$) migratory speeds plotted as a function of radial position within the spheroid body. As shown in the schematic, the quantity $tan(\Theta) = |\boldsymbol{v_t}|/|\boldsymbol{v_r}|$ measures the magnitude of the tangential component of migratory speed with respect to radial speed. While in 2 mg/ml collagen such ratio remains constant, it decreases progressively in 4 mg/ml, thus suggesting that invasion at the spheroid periphery is directed predominatly in the radial direction. Overall, these results suggest that high ECM confinement induces collective invasion in MDA-MB-231 spheroids which is chracaterized by more regular cell shapes and slower cell motions at the invasive periphery, where cells display highly coordinated and predominantly radial motions. Taken together, these results implicate that a confinement-induced jamming transition takes place as MDA-MB-231 spheroids invade collectively into high density collagen.

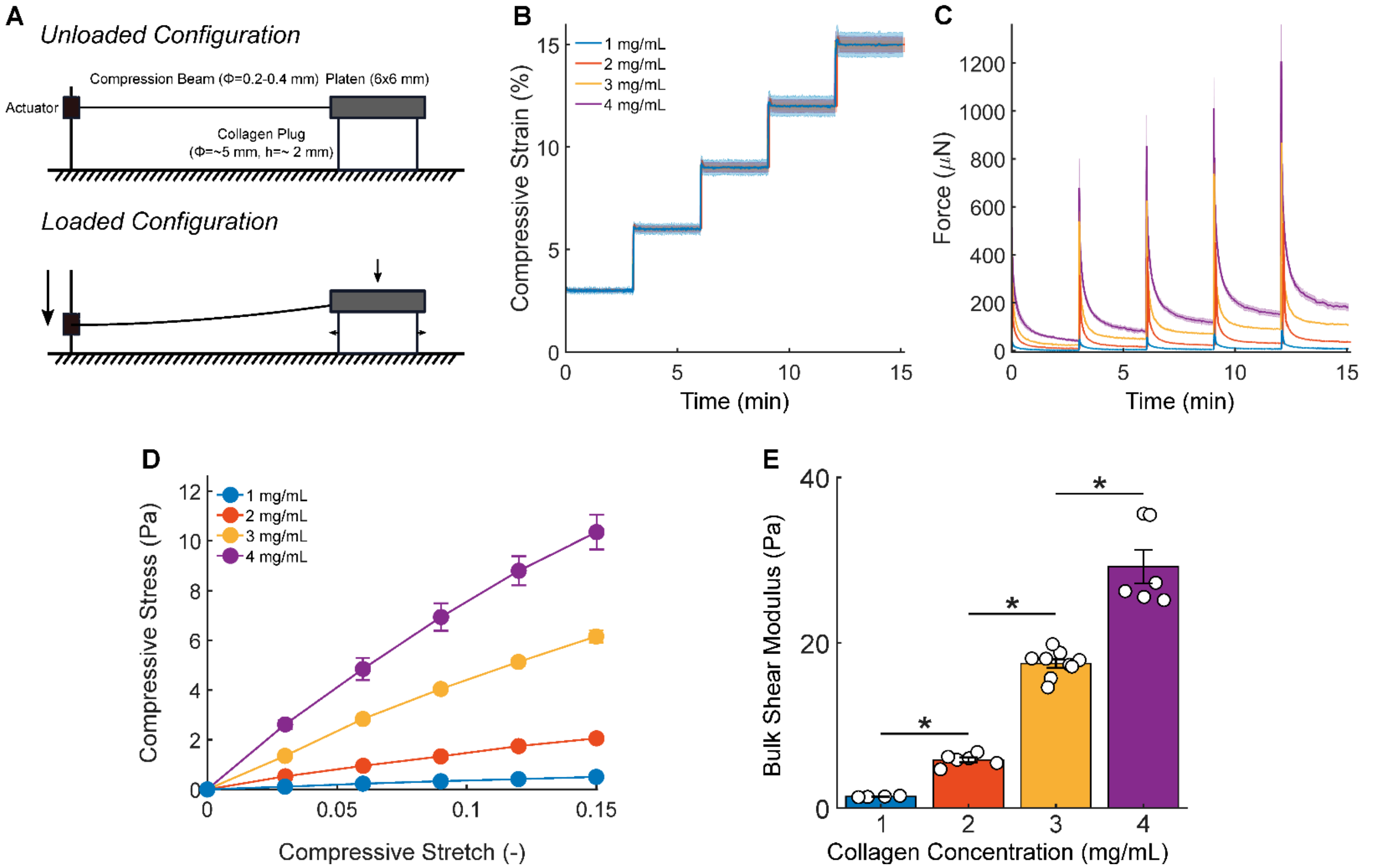

Figure S8. Mechanical characterization of collagen hydrogels. Related to Figure 4. Cylindrical collagen plugs at concentrations of 1-4 mg/ml were subjected to uniaxial unconfined compression by means of a microbeam connecting the piezoelectric actuator to the compression platen. (**A**). Schematic illustration of the microscale system used for testing the mechanical properties of acellular collagen gels (5mm in diameter and 2mm in height). It should be noted that, upon loading, the reaction force generated by the compressed collagen gel was calculated by measuring the deflection of the microbeam tracked using a digital camera. Preliminary tests allowed us to determine the optimal diameter (Φ) of the microbeam that maximized force resolution for 1 mg/ml (Φ = 203.2 μm), 2 mg/ml (Φ = 304.8 μm), and 3-4 mg/ml (Φ = 406.4 μm) collagen gels. (**B**) Unconfined compression tests were carried out in 5 incremental steps, each with a magnitude equal to 3% of the original height of the sample, interspersed with 3-minute long hold durations. (**C**) The resultant force increased steadily with increasing concentration of collagen while showing a highly reproducible behavior. For each sample, the equilibrium force-displacement data was converted into a compressive stress vs. compressive stretch plot (**D**) which was then fitted to a neo-Hookean material model (STAR Methods) to obtain the value of the bulk shear modulus (**E**). The bulk stiffness increased progressively with increasing collagen concentration. Mechanical data are shown from 1 mg/ml (n = 4), 2 mg/ml (n = 6), 3 mg/ml (n = 9), and 4 mg/ml (n = 6) collagen gels. Data are presented as mean ± SEM and * indicates statistical significance at $p < 0.05$.

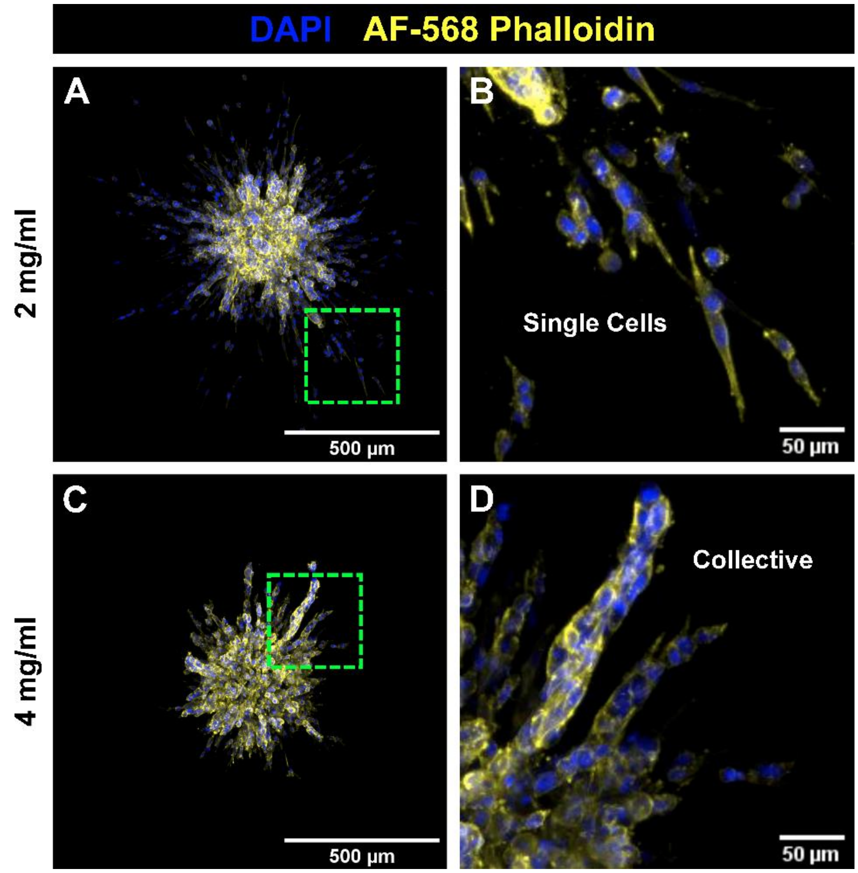

Figure S9. Cytoskeletal structure of MDA-MB-231 cells within invasive tumor spheroids. Related to STAR Methods. Fluorescent staining (maximum intensity projections) of F-actin in MDA-MB-231 spheroids embedded in 2 mg/ml (**A,B**) and 4 mg/mL (**C,D**) collagen. DAPI staining is shown in blue while AlexaFluor 568 (AF-568) Phalloidin is shown in yellow. The right column provides zoomed-in views of the green dashed boxes and serves to highlight single cells and collective protrusions invading, respectively, in low- and high-density collagen. A recent study (Grosser et al., 2021) proposed that the formation of supracellular actin cortex can enable cell clusters to migrate as coordinated units. In our system, F-actin staining did not reveal differences in the cytoskeletal organization of cells invading in low (2 mg/ml) and high (4 mg/ml) collagen density. Instead, we observed elongated cells invading individually in low density collagen, while cells packed in high density collagen invaded as a multicellular collective under physical confinement from the ECM.

SUPPLEMENTARY TABLES

Table S1: Increasing collagen density does not impact cell proliferation or spheroid growth rate. Related to Figures 2 and 3. Both types of macro-spheroids (MCF-10A and MDA-MB-231) in both low and high collagen densities (2 and 4 mg/mL) were formed starting from similar cell numbers at initial seeding (STAR Methods). Following 48 hours of spheroid formation and additional 48 hours of invasion in collagen, spheroids were fixed, optically cleared and imaged. Cell numbers within each macro-spheroid were counted using our custom cell identification algorithm (cf. STAR Methods, Figure S2). Spheroid radius was calculated from segmentation of DIC time-lapse movies to obtain the main spheroid area over time as $radius = \sqrt{area/\pi}$. Radial growth rate is estimated from linear fitting of the spheroid radius over time. Increasing collagen density does not impact either cell count or radial growth rate while it affects the mode of migration (cf. Figures 2 and 3).

Cell type	Collagen concentration	Cell count	p-value	Radial growth rate (μm/hr)	p-value	n
MCF-10A	2 mg/ml	10369 ± 509	0.51	1.22 ± 0.16	0.24	3
	4 mg/ml	10679 ± 536		1.16 ± 0.12		3
MDA-MB-231	2 mg/ml	6230 ± 793	0.19	0.94 ± 0.27	0.53	3
	4 mg/ml	5536 ± 331		1.06 ± 0.15		3

Table S2. A sensitivity analysis shows that phase diagram is not sensitive to the threshold values used to define the different material phase states. Related to Figure 5. Phase states of spheroids were determined from thresholding on distribution of simulated distance of the outermost 5% cells in the spheroid. Distances below the 34th percentile, between the 34th and 64th percentile, and above the 64th percentile lead to classification of migrating cells, repectively, as solid-like, fluid-like, and gas-like. We performed a sensitivity analysis to assess the dependence of the resulting phase diagram on the threshold values used to define the phase states, by applying ±2.5% changes to such thresholds. Results were quantified as percent change (%) in the number of points that have switched phase states (for example: solid to liquid, liquid to gas, etc.) from the total number of points in the phase diagram, due to the different threshold combinations. Our sensitivity analysis shows that the phase diagram is not significantly impacted by the exact values of threshold used to define the material states, with less than 5% of points changing material phase in correspondence of the highest change in threshold values tested.

Gas Phase Threshold (%)	Liquid Phase Threshold (%)					
	31	32	33	34	35	36
62	4.872%	3.590%	2.821%	2.051%	2.051%	3.333%
63	3.077%	3.077%	2.051%	1.026%	1.026%	2.308%
64	3.077%	2.051%	1.026%		0.000%	1.795%
65	4.872%	2.821%	1.795%	0.769%	0.769%	2.564%
66	4.872%	3.846%	2.821%	1.795%	1.795%	3.590%
